# Granular layEr Simulator: Design and Multi-GPU Simulation of the Cerebellar Granular Layer

**DOI:** 10.3389/fncom.2021.630795

**Published:** 2021-03-16

**Authors:** Giordana Florimbi, Emanuele Torti, Stefano Masoli, Egidio D'Angelo, Francesco Leporati

**Affiliations:** ^1^Custom Computing and Programmable Systems Laboratory, Department of Electrical, Computer and Biomedical Engineering, University of Pavia, Pavia, Italy; ^2^Neurocomputational Laboratory, Neurophysiology Unit, Department of Brain and Behavioral Sciences, University of Pavia, Pavia, Italy; ^3^Istituti di Ricovero e Cura a Carattere Scientifico (IRCCS) Mondino Foundation, Pavia, Italy

**Keywords:** computational modeling, neuroscience, granular layer simulator, graphics processing unit, high performance computing, parallel processing

## Abstract

In modern computational modeling, neuroscientists need to reproduce long-lasting activity of large-scale networks, where neurons are described by highly complex mathematical models. These aspects strongly increase the computational load of the simulations, which can be efficiently performed by exploiting parallel systems to reduce the processing times. Graphics Processing Unit (GPU) devices meet this need providing on desktop High Performance Computing. In this work, authors describe a novel Granular layEr Simulator development implemented on a multi-GPU system capable of reconstructing the cerebellar granular layer in a 3D space and reproducing its neuronal activity. The reconstruction is characterized by a high level of novelty and realism considering axonal/dendritic field geometries, oriented in the 3D space, and following convergence/divergence rates provided in literature. Neurons are modeled using Hodgkin and Huxley representations. The network is validated by reproducing typical behaviors which are well-documented in the literature, such as the center-surround organization. The reconstruction of a network, whose volume is 600 × 150 × 1,200 μm^3^ with 432,000 granules, 972 Golgi cells, 32,399 glomeruli, and 4,051 mossy fibers, takes 235 s on an Intel i9 processor. The 10 s activity reproduction takes only 4.34 and 3.37 h exploiting a single and multi-GPU desktop system (with one or two NVIDIA RTX 2080 GPU, respectively). Moreover, the code takes only 3.52 and 2.44 h if run on one or two NVIDIA V100 GPU, respectively. The relevant speedups reached (up to ~38× in the single-GPU version, and ~55× in the multi-GPU) clearly demonstrate that the GPU technology is highly suitable for realistic large network simulations.

## Introduction

The challenge to understand, reproduce and simulate the human brain activities needs more and more High-Performance Computing (HPC) support, in particular, where heterogeneous elements, described by complex mathematical models, have to be simulated as fast as possible (Bouchard et al., 2016). For example, computational modeling in neuroscience has to perform large-scale simulations to reproduce complex physiological behaviors of neuronal networks. Among the different aspects that ask for HPC in neuroscience, some have a more relevant impact as the network dimension, i.e., the number of neurons, and the connections to model. Nowadays, several research groups work on reproducing the functionalities of very large areas of the brain (Beyeler et al., 2014; Cremonesi and Schürmann, 2020). To this aim, they need multicore and/or manycore technologies capable of reducing the processing time and of ensuring the power, memory, and storage capabilities offered by HPC solutions (Fidjeland et al., 2013). Another aspect to consider is the model to use for the neuron representation and the detailed morphologies introduced in the network. Starting from the simple Leaky Integrate and Fire (LIF) model up to the more complex Hodgkin Huxley (HH) one, all the mathematical representations are characterized by a variable number of differential equations, which strongly increases the computational load of the simulations (Izhikevich, 2004). Moreover, the detailed morphologies provide information about how to perform the signal exchange between neurons in the network and how the potential evolves inside the single element. If not properly managed, those aspects can easily increase the computational load of the simulation. A further issue to consider is the duration of the neuronal activity to reproduce. Particular attention should be given to the time integration step that directly determines the number of times that the differential equations have to be solved.

Recently, the number of research neuroscience groups using multicore and/or manycore architectures has indeed increased due to the need of high computing power to simulate complex and realistic neuronal models. Among HPC architectures, the Graphics Processing Unit (GPU) technology is becoming one of the most popular since it is capable of processing in parallel the neuronal activity of a huge number of cells. One important aspect that can make the GPUs useful in this research field is that they can be hosted in desktop systems as well as in supercomputers.

In this work, authors present the Granular layEr Simulator (GES), a system capable of reconstructing in detail the granular layer network of the cerebellum (a major cortical structure of the vertebrate brain) in a 3D space and of reproducing its neuronal activity. This code has been written in C language and using the OpenMP API together with the CUDA framework to efficiently exploit desktop architectures characterized by multicore CPUs connected to single and multi-GPUs. The simulator consists of three modules: the *network design* displaces the neurons and the glomeruli in a volume and connects them considering axonal/dendritic field geometries, oriented in the 3D space, and following the convergence/divergence rates that, to the best of the authors' knowledge, are the most relevant in the literature. Once the neurons displacement and connections have been elaborated, the *simulation* module can reproduce the network neuronal activity. The neurons are modeled using the Hodgkin and Huxley representation (Hodgkin and Huxley, 1990). The third module is the *graphical interface* that allows the user to generate several network configurations, to simulate and to graphically visualize them in a 3D space. In fact, the aim is to build a parametric network that can reproduce different configurations only by changing the values of suitable variables. This parametric system is also scalable allowing the reproduction of networks with different sizes.

Section Materials and Methods presents the granular layer model and the optimized codes developed for the network reconstruction. Moreover, the serial and parallel codes of the simulation module will be detailed. Then, the graphical user interface will be described. Section Results and Discussion presents the results and an exhaustive analysis of the neurons placement with relative connections, of the processing times and of the system memory occupancy. A comparison with the state of the art and possible future works are shown. Finally, section Conclusions draws the main conclusions and the possible future directions of the work.

## Materials and Methods

In the previous works, authors developed the simulators for the single cells hosted in the cerebellar granular layer. The neurons have been modeled exploiting the Hodgkin and Huxley representation and their simulators have been developed targeting parallel devices. This activity had a crucial importance to validate the neuronal behaviors and to evaluate the best parallel technologies to use in the network implementation (Florimbi et al., 2016, 2019).

The development of the GES required three main phases that represent a further step-on in the conducted research. At first, an efficient algorithm to reconstruct the granular layer network in a 3D space was developed. It places and connects different types of neurons as realistically as possible, taking into account their cellular morphology and their axonal/dendritic field geometries, oriented in the 3D space. The cells connections are the input of the second step, which concerns the neuronal activity reproduction of the layer. The network activity simulation has been carried out on one of the most recent multi-GPUs systems, in order to reduce the computational time. In the last phase of the work, a graphical interface has been developed to visualize the displacement, the connections and the activation patterns of the different neurons providing to the scientists a useful and easy tool that allows to setup the simulation and to monitor its own behavior.

### Overview of the Cerebellar Granular Layer Model

#### The Neurons Models

The Hodgkin-Huxley (HH) model (Hodgkin and Huxley, 1990) is one of the most accurate and complex representations to reproduce the neuronal activity. The model describes the cellular membrane as a capacitor *C*_*m*_ since it keeps the ions separated on its sides. The capacitor is connected in parallel with different branches, each one including a resistor and a voltage generator connected in series. The resistors stand for the ionic channels, contained in the membrane, which allow the ions crossing. The voltage generators represent the active transport mechanisms that characterized the cellular activity. The current *I* flowing through the membrane is described as in Equation (1):

(1) $$\begin{array}{l} I=\;C_{m}\frac{dV_{m}}{dt}+I_{syn}{+\;I}_{ions} \end{array}$$

where *V*_*m*_ is the membrane potential, *I*_*syn*_ the synaptic current, and *I*_*ions*_ is the sum of the ionic currents. Each ionic current (*I*_*ion*_) is defined as the product between the channel conductance *g*_*ion*_ and the difference between the membrane potential *V*_*m*_ and the equilibrium potential of the specific ion *E*_*ion*_ (Equation 2):

(2) $$\begin{array}{l} I_{ion}=g_{ion}\left(V_{m}-E_{ion}\right) \end{array}$$

The ionic channels are characterized by the presence of *gating particles*, whose position inside the channel allows its opening or closure. The HH model reproduces how they dynamically affect the channel conductance as in Equation (3):

(3) $$\begin{array}{l} g_{ion}={\bar{g}}_{ion}\times x_{ion}^{z}\times y_{ion}^{k} \end{array}$$

where ${\bar{g}}_{ion}$is the maximum conductance of the channel, *x*_*ion*_ and *y*_*ion*_ are the probabilities that the gating particles occupy a certain position in the membrane. *z* and *k* represent the number of activation and inactivation particles for each channel (Florimbi et al., 2017). The probability of a particle of being in a permissive state depends from two coefficients *α*_*n*_ and *β*_*n*_ related to the velocity of transition (D'Angelo et al., 2001). The relation is given by:

(4) $$\begin{array}{l} \frac{dn}{dt}=\alpha_{n}\left(1-n\right)-\beta_{n}n \end{array}$$

where the probability of being in a permissive state is *n*. Equation (4) can be simplified using these two relations:

(5) $$\begin{array}{l} n_{\infty }=\frac{\alpha_{n}}{\alpha_{n}+\beta_{n}} \end{array}$$

(6) $$\begin{array}{l} \tau_{n}=\frac{1}{\alpha_{n}+\beta_{n}} \end{array}$$

where *n*_∞_ and *τ*_*n*_ are the stationary part and the activation time of the channel. Equation (4) can then be rewritten as:

(7) $$\begin{array}{l} \frac{dn}{dt}=\frac{n_{\infty }-n}{\tau_{n}} \end{array}$$

which is solved by:

(8) $$\begin{array}{l} n\left(t\right)=n_{\infty }-\left(n_{\infty }-n_{0}\right)e^{-\frac{t}{\tau_{n}}} \end{array}$$

where *n*_0_ is the initial value of *n*.

The final model is obtained considering the gating particles for each ionic channel and including these relations in Equation (1):

(9) $$\begin{array}{l} I=C_{m}\frac{dV_{m}}{dt}+I_{syn}+{\bar{g}}_{k}n^{4}\left(V_{m}-V_{k}\right)+{\bar{g}}_{Na}m^{3}h\left(V_{m}-V_{Na}\right)\\ \;+\;{\bar{g}}_{L}\left(V_{m}-V_{L}\right) \end{array}$$

where *n* is the gating particle of the potassium channel and *m* and *h* are the ones of the sodium channel.

Concerning the soma of the granule (GRCs), the model described in D'Angelo et al. (2001), takes into account some particular mechanisms related to ions. The sodium channel is represented by three currents: a fast Na^+^ (I_Na−f_), a persistent Na^+^ (I_Na−p_), and a resurgent Na^+^ (I_Na−r_) currents. The potassium channel is represented by five currents that reproduce different dynamics: a current for rectified delayed channels (I_K−V_), one depending on intracellular calcium concentration (I_K−Ca_), one for inward rectified channels (I_K−IR_), one for type-A channels (I_K−A_) and a current for slow kinetic channels (I_K−slow_). The reversal or Nernst potential of the sodium and of the potassium channels are constant during the neuronal activity evaluation. The calcium channel is characterized by a variable intracellular calcium concentration. The Ca^2+^ dynamic is described by the following differential equation (Florimbi et al., 2016) (Equation 10):

(10) $$\frac{d[Ca^{2+}]}{dt}=\frac{-I_{Ca}}{2FAd}-(\beta_{Ca}\left(\left[Ca^{2+}\right]-{\left[Ca^{2+}\right]}_{0}\right))$$

where *d* is the depth of the vesicle linked to the cellular membrane, whose area is indicated with *A*. *β*_*Ca*_ determines the calcium ions leakage from the cell, *F* is the Faraday constant, *[Ca*^2+^*]*_0_ is the calcium concentration at rest. Once *[Ca*^2+^*]* is computed, *E*_*ca*_ can be determined and used in the calcium current evaluation. The kinetic of these ionic channels is described using the HH model and the gating particle mechanism described above. Each channel is characterized by a different number of activation and inactivation particles.

Also for the Golgi (GOC), the model adopted to reproduce its activity considers several ionic currents (Solinas et al., 2008; D'Angelo et al., 2013). The ones that reproduce the regular pacemaking of the cell are the sodium persistent current (I_Na−p_), the h current (I_h_), the SK-type small conductance calcium-dependent potassium current (I_K−AHP_) and the slow M-like potassium current (I_K−SLOW_). These currents together with the sodium resurgent one (I_Na−r_) and the A-current (I_K−A_) regulate the response frequency and delay the fast phase of a spike, which is present after the hyperpolarization. The I_Ca−LVA_ reinforces the rebound depolarization. The rebound excitation is caused by the currents I_Ca−LVA_ and I_h_. All these currents are described by the gating particles model explained before. Instead, the I_K−AHP_ current is simulated with a Markov gating scheme characterized by six states, four closed and two open (Solinas et al., 2008; Florimbi et al., 2019).

#### The Synapses Models

The term *I*_*syn*_ in Equation (4) represents the synaptic currents, i.e., the currents injected to the cell by their connected neurons through excitatory and inhibitory synapses. To compute the synaptic current, it is important to provide a model that reproduces the presynaptic and the post-synaptic terminals. In the first case, a three-state kinetic scheme has to be solved to compute the amount of neurotransmitter (T) released by the presynaptic terminal (Nieus et al., 2006). This neurotransmitter reaches the receptors hosted in the post-synaptic terminal, reproduced by a model that allows to compute the currents that flow in the receptors channels. The excitatory synapses is characterized by the N-methyl-D-aspartate (NMDA) and the *α*-amino-3-hydroxy-5-methyl-4-isoxazolepropionic acid (AMPA) receptors in the post-synaptic terminal (Nieus et al., 2006), while the inhibitory synapses present the gamma-Aminobutyric acid (GABA) one (Nieus et al., 2014). The current flowing in each receptor channel is computed solving a kinetic scheme of first-order reactions, with five (NMDA), three (AMPA) and eight (GABA) states. A detailed description of the dynamic of these receptors can be found in Nieus et al. (2006) for NMDA and AMPA, and in Nieus et al. (2014) for GABA.

Concerning AMPA receptors, there are three possible channel states: open (O), closed (C), and desensitized (D). Therefore, the current contribution is given by:

(11) $$\begin{array}{l} I_{AMPA}=g_{\max,AMPA}\left(V_{m}-V_{rev,AMPA}\right)O\left(T\right) \end{array}$$

where *g*_max,*AMPA*_ is the maximum conductance of the AMPA receptor (1,200 pS), *V*_*m*_ is the membrane potential, *V*_*rev, AMPA*_ is the ionic reversal potential and *O*(*T*) is the probability of being in the open state, which depends from the concentration of neurotransmitter *T*.

The NMDA receptor is more complex since it has five possible states: three closed states (C1, C2, and C3), an open state (O), and a desensitized state (D). The current contribution is given by:

(12) $$\begin{array}{l} I_{NMDA}=g_{\max,NMDA}\left(V_{m}-V_{rev,NMDA}\right)O\left(T\right)B \end{array}$$

where *g*_max,*NMDA*_ is the maximum conductance of the NMDA receptor (18,800 pS), *V*_*m*_ is the membrane potential, *V*_*rev,NMDA*_ is the ionic reversal potential, *O*(*T*) is the probability of being in the open state and B is a term to take into account the concentration of the Mg^2+^ ion.

The GABA inhibitory receptors are made up of two parts, called *α*1-GABA and *α*6-GABA. These two parts can be modeled using the same Markov chain, which is made up of two open states (OA1 and OA2), three closed states (C, CA1, and CA2) and three desensitized states (DA1, DA2, and DA2f).

The current of each part of the GABA receptor is given by

(13) $$\begin{array}{l} I_{GABA}=g_{\max,GABA}\left(V_{m}-V_{rev}\right)\left(OA1\left(T\right)+OA2\left(T\right)\right) \end{array}$$

where *g*_max,*GABA*_ is the maximum conductance (918 pS for *α*1-GABA and 132 pS for *α*6-GABA), *V*_*m*_ is the membrane potential, *V*_*rev,GABA*_ is the ionic reversal potential and the sum OA1(T)+OA2(T) represents the probability of being in an open state.

Finally, *I*_*syn*_ is given by receptor currents (Equation 14):

(14) $$\begin{array}{l} I_{syn}=I_{NMDA}+I_{AMPA}+I_{GABA} \end{array}$$

A deeper description of the GRC, GOC, and synaptic models can be found in Florimbi et al. (2016, 2019).

#### The Network Connectivity

**Figure 1:**
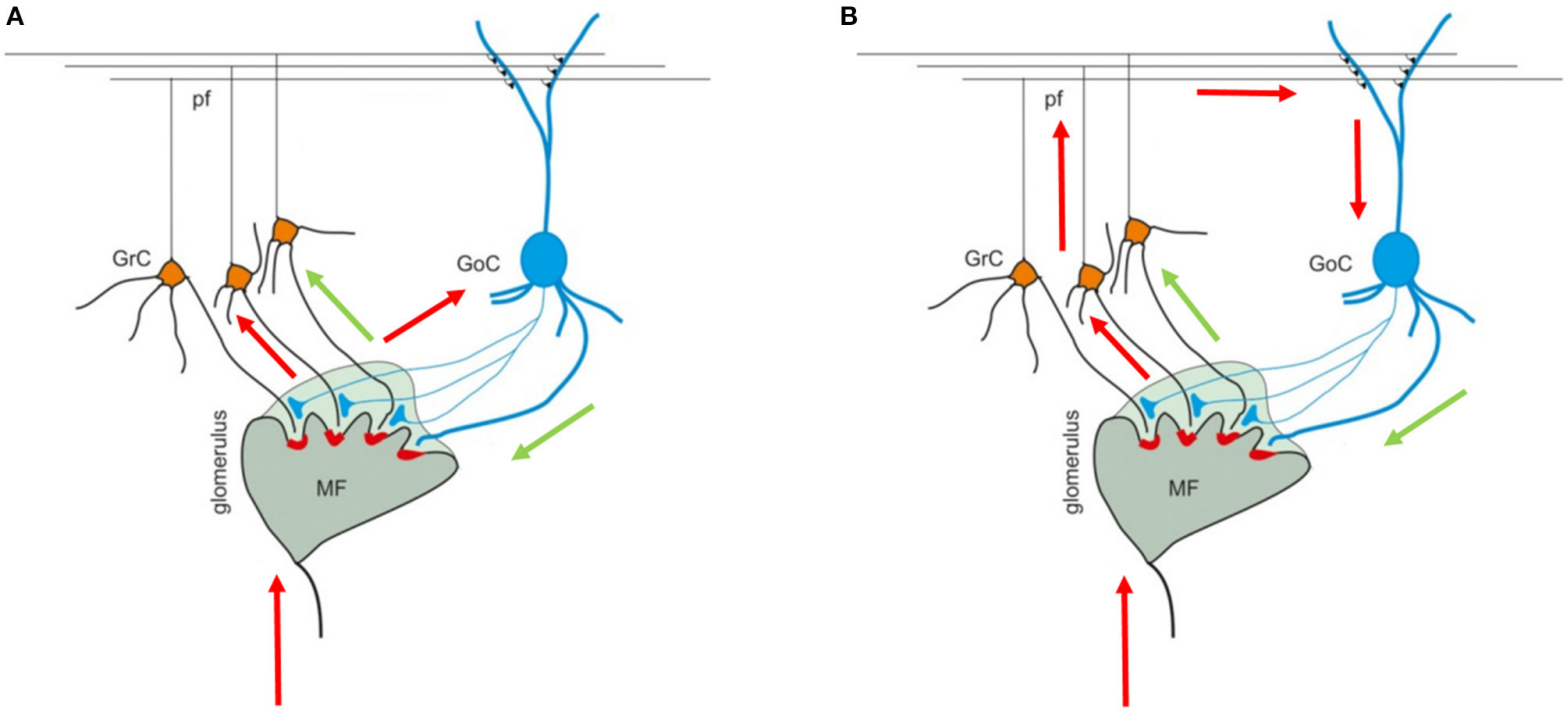
Cerebellar granular layer. **(A)** The granular layer circuit receives the input from the mossy fibers (MFs) that reach the glomeruli (GLOs). Here, they reach and excite the Golgi cells (GOCs) and granules (GRCs) dendrites. Once the GRCs are stimulated, the signal travels along the GRCs ascending axon and parallel fibers (PFs) and, then, can reach the GOCs apical dendrites (feedback loop). **(B)** On the other hand, the MFs signals reach the GOCs cells that inhibit the GRCs (feedforward loop). The black arrows indicate the direction of the signals in the loops. The image is taken from Mapelli et al. (2014).

The cerebellar cortex is composed of three layers (the *granular*, the *Purkinje*, and the *molecular layers*), each one including different types of neurons. The granular layer hosts GRC and GOC cells that connect their dendrites and axons in structures called glomeruli (GLOs), reached also by the mossy fibers (MFs). These elements are connected in the so called *feedforward* and *feedback* loops (Figures 1A,B) (Mapelli et al., 2014). In the first case, the MFs excite the GRCs and GOCs dendrites and these latter inhibit the GRCs; in the second case, the MFs excite the GRCs and, then, the parallel fibers (PFs) excite the GOCs that inhibit the GRCs.

All the elements (GOC, GRC, GLO, MF, and PF) are connected following convergence/divergence rules present in literature. According to Solinas et al. (2010) and D'Angelo et al. (2016), the convergence rate between GLOs and GRCs is 4:1, which means that 3–5 GRC's dendrites are connected to each GLO. The GRCs dendrites cannot reach GLOs located more than 40 μm away (the mean dendritic length is 13.6 μm) and a single GRC cannot send more than one dendrite into the same GLO (D'Angelo et al., 2013). Moreover, each GRC must project its dendrites to four different GLOs (Solinas et al., 2010). Each GLO has about 50 connections available for the GRCs dendrites since the divergence rule between GLOs and GRCs dendrites is 1:53 (D'Angelo et al., 2016).

The GOCs axons are placed in the granular layer spreading longitudinally. They enter in the GLOs to inhibit the GRCs. The convergence rate between GLOs and the GOCs is 50:1 (Solinas et al., 2010; D'Angelo et al., 2016). A GOC axon can enter only in GLOs without GRCs in common: a GRC is not inhibited twice by the same GOC (Solinas et al., 2010). Moreover, each GOC axon can reach and inhibit a maximum of 40 different GLOs (i.e., reaching ~2,000 GRCs following the ratio GOCs:GRCs equal to 1:430) (Korbo et al., 1993; D'Angelo et al., 2013).

The GOCs basal dendrites spread around the soma on the same plane. They reach the GLOs where they make excitatory synapses with the MFs. Each GOC receives excitatory inputs from about 40 MFs on basal dendrites (Kanichay and Silver, 2008; D'Angelo et al., 2013).

The GRCs axons cross vertically the cerebellar Purkinje layer (i.e., ascending axon), which contains the Purkinje soma, and reach the molecular layer where it branches into PFs running transversally. It has been observed that GRCs form their connections through PFs and also along the ascending axon (D'Angelo et al., 2013). Moreover, D'Angelo et al. (2016) report that the convergence rate between the ascending axon and the GOCs is 400:1 and between the PFs and the GOCs is 1,000:1. GOCs are connected through gap-junctions present in the basal and apical dendrites (D'Angelo et al., 2013, 2016).

### The Granular layEr Simulator

The basic idea followed in the network development has been to construct a *non-fixed* parametric structure. This means that even though the network is defined by specific structural and connections rules, it is still possible to modify its size, reproducing different configurations. The volume that will be reproduced in this work is 600 (*length*) × 150 (*height*) × 1,200 (*depth*) μm^3^. This flexibility should be intended only in terms of parameters variability rather than new constraints introduction. The serial algorithm developed to reconstruct the granular layer is written in C language, which allows direct and efficient dynamic memory management.

#### Network Design

The network design module performs two main operations: the elements displacement and connection in a 3D volume.

In this case, the serial algorithm starts computing the number of GOCs, GRCs, and GLOs, referring to typical rat densities as shown in Table 1. It also shows the number of elements considered in the network configuration under study. Finally, since the neurons soma is modeled as a sphere, the correspondent diameter is reported.

**Table 1:** GOCs, GRCs, and GLOs density values and the number of elements that a volume of 600 × 150 × 1,200 μm^3^ can host.

**Cell or element ID**	**Density (mm^–3^)**	**Cells (or element) number**	**Soma diameter [μm]**
GOCs	9,000 (Korbo et al., 1993)	972	15 (Dieudonné, 1998; Houston et al., 2017)
GRCs	4,000,000 (Korbo et al., 1993)	432,000	5 (Solinas et al., 2010)
GLOs	300,000 (Korbo et al., 1993)	32,400	5 (Rossi and Hamann, 1998)

*Moreover, soma diameters are considered*.

**Figure 2:**
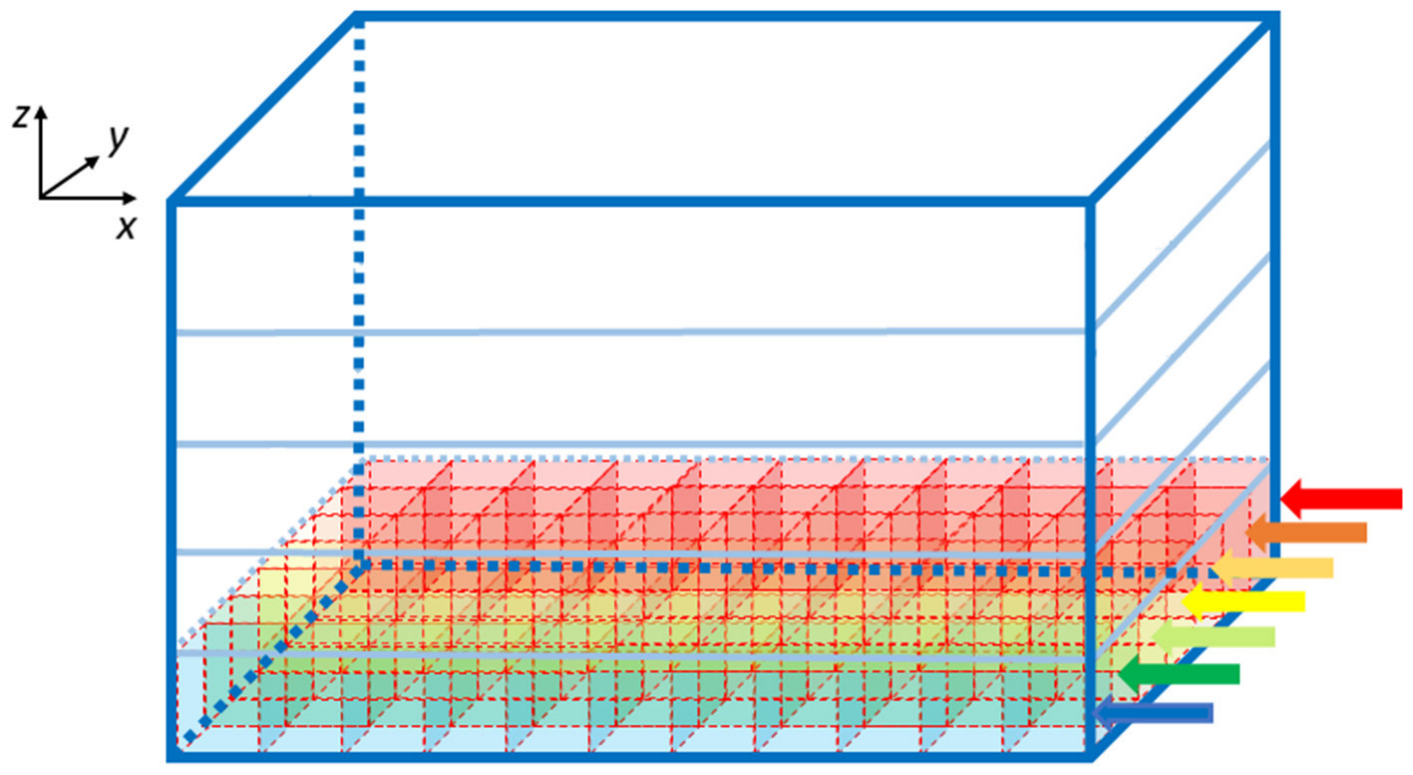
Volume division for the displacement of the GOCs. The volume is divided into z-layers (five in this image). Each z-layer (x-y plane) is divided into rows along the y-axis (indicated with the arrows), where rectangular parallelepipeds are placed to host the cells. The algorithm starts placing the neurons from the blue row to the red one. The procedure is repeated for each z-layer.

Once the elements number is known, the algorithm computes the GOCs, GRCs and GLOs coordinates that are stored in three arrays (*c_goc, c_grc*, and *c_glo*, respectively), dynamically allocated through the *malloc* routine in the initial phase of the code. The algorithm inserts the elements in the space in a *partially random* way, considering specific physiological requirements (Korbo et al., 1993; Dieudonné, 1998; Rossi and Hamann, 1998; Solinas et al., 2010). The height of the volume (*z-axis*) is divided into several layers, whose number is related to the dimensions of the GOCs since they are the first type of elements introduced in the volume. Each layer includes several boxes, organized in rows (shown by the arrows in Figure 2), which dimensions are related to the GOCs ones. The basis of the box is a square, whose side is equal to the GOC diameter. On the other hand, the height of the box is higher than the diameter so that the algorithm can randomly compute the *z* coordinate of the cell inside the box. In this way, all the GOCs inside a layer are not placed at the same height. The algorithm has to insert a defined number of GOCs in each layer, selecting a box for each cell. The algorithm chooses the suitable box following morphological constraints (i.e., dendrites length and depth) and avoiding boxes already occupied by other neurons. When a cell is placed in a box, its *x* and *y* coordinates are defined. Once all the GOCs have been placed in the volume, the algorithm inserts the GLOs and, then, the GRCs, both represented by spheres with 5 μm diameter (Table 1). The strategy adopted to place GLOs and GRCs is the same as for GOCs. During the GLOs and GRCs displacement, a further constraint is added to avoid that these elements overlap the GOCs. Finally, the correspondent coordinates are written in the *c_goc, c_glo*, and *c_grc* arrays.

Once the elements have been placed in the volume, the algorithm starts to connect them, generating the *connection matrices*. They are linear arrays containing the information on how elements are connected following convergence/divergence rules. The *connection matrices* reproducing the feedforward and feedback loops (D'Angelo et al., 2016) contain the links among the following elements (the name of the *connection matrices* is reported between brackets):

- GRCs and GLOs (*link_grc_glo*);
- GOCs axon and GLOs (*link_goca_glo*);
- GOCs basal dendrites and GLOs (*link_gocdb_glom*);
- GRCs (ascending axon and PFs) and GOCs (*aa_goc_link, pf_goc_link*);
- MFs and GLOs (*mf_glom_clustering*);
- GOCs and GOCs (*gap_junction*).

For each type of connection, the authors developed a suitable algorithm capable of connecting the elements following the morphological rules and the convergence/divergence ratios.

As an example, the algorithm that links the GRCs to the GOCs through ascending axon and PFs is detailed in the following.

**Figure 3:**
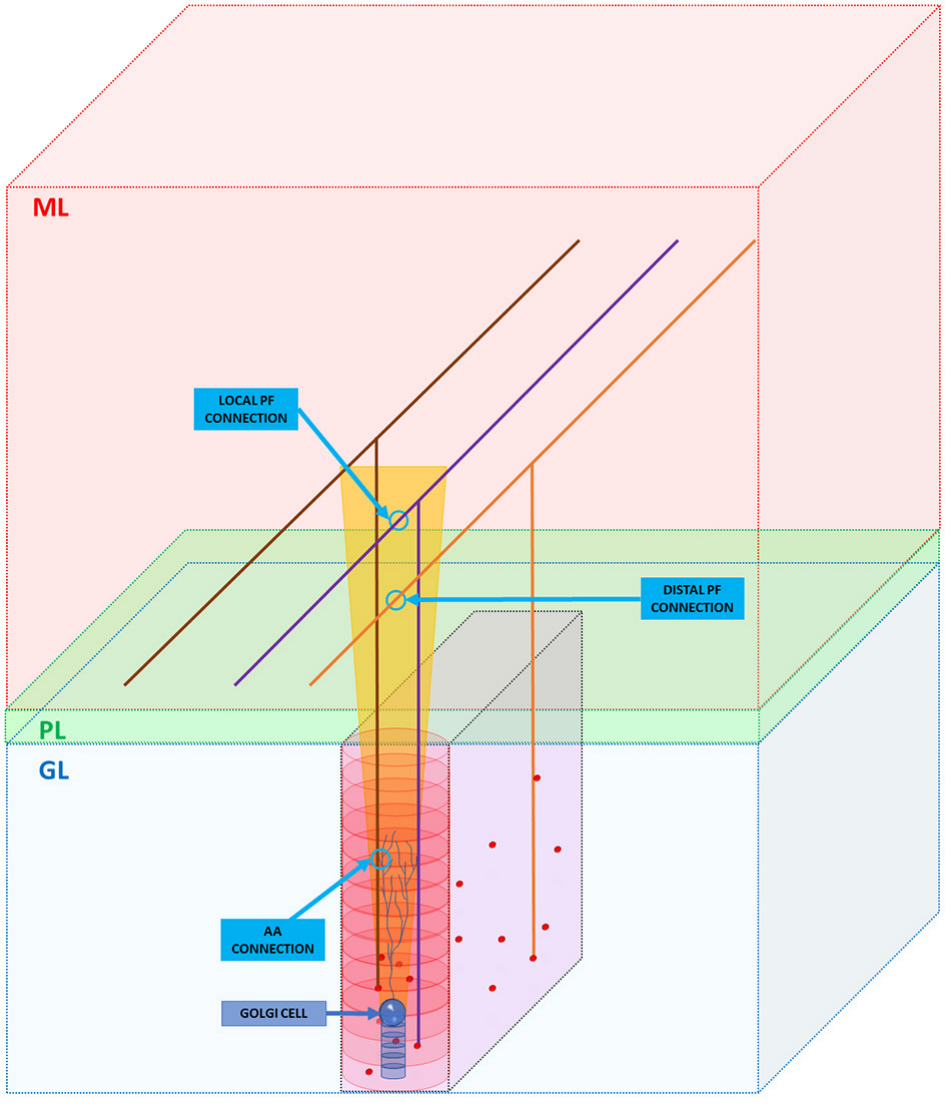
GRC ascending axon and PF connections with GOC. The figure shows the different layers of the cerebellum cortex: granular layer (GL), Purkinje layer (PL), and molecular layer (ML). In the GL, GRC soma, and GOC soma are represented with red and blue spheres, respectively. The yellow trapezoid represents a schematization of the area occupied by the GOC apical dendrites (partially shown inside the area). The image shows three examples of connection. Firstly, the algorithm connects the GOC apical dendrites with the GRC ascending axon (AA CONNECTION). Then, it performs the connections through PFs (LOCAL PF CONNECTION and DISTAL PF CONNECTION). Notice that in the image, the sizes do not scale proportionally to improve the graphical view.

As said in section Overview of the Cerebellar Granular Layer Model, the GRCs axons cross vertically the cerebellar Purkinje layer and reach the molecular one where it branches into PFs running transversally, i.e., along the *y*-axis (Figure 3). Even if this work aims to reproduce the granular layer, it is important to take into account these connection schemes, in order to reproduce the feedback and feedforward loops, simulating all the connections between neurons. As previously said, the GRCs form their connections with GOCs through PFs and also along the ascending axon with a convergence rate of 1,000:1 and 400:1, respectively (D'Angelo et al., 2016) (D'Angelo et al., 2013). Firstly, the algorithm computes the connections between the ascending axons and the GOCs. When the algorithm has to find those GRCs to connect to, it builds an elliptical cylinder (in red in Figure 3) around the GOC soma, whose major axis is the maximum length of the apical dendrites, while the minor axis is given by their depth. For each GOC, the algorithm selects 400 GRCs inside the red cylinder avoiding the area under the GOC soma, denoted by a blue cylinder in Figure 3, where it is less probable to have connections. The algorithm randomly selects a GRC among the 400, and checks if it is inside the red cylinder. If yes, the connection is performed (AA CONNECTION in Figure 3) and the GRC index is stored in the linear matrix *aa_goc_link*. Considering the PFs, the GOC receives 400 connections through the PFs of local GRCs and 1,200 distal connections (D'Angelo et al., 2013). The algorithm selects the GOCs to link to the GRCs, checking if in the red cylinder there are GRCs, whose PF crosses the apical dendrites area (as in the purple case in Figure 3): if yes, the connection is made (LOCAL PF CONNECTION). If the PF that crosses the apical dendrite area belongs to a GRC far from the GOC soma (orange parallel fiber), a distal connection (DISTAL PF CONNECTION) is implemented. These connections are stored in the linear matrix *pf_goc_link*.

Another interesting part of the system performs the connections between MFs and GLOs. In this case, authors developed a custom clustering algorithm to meet physiological constraints. Authors in Sultan and Heck (2003) described how the MFs branch in the cerebellum. They form clusters of presynaptic enlargements called *rosettes*, which represent the presynaptic part in the GLOs. Each MF can form multiple clusters of rosettes arranged with a characteristic spatial organization. In particular, authors in Sultan and Heck (2003) demonstrate that the clusters belonging to the same MF are separated from each other. For this reason, given the dimension of the network considered in this work, it is not reasonable to find two clusters belonging to the same MF.

They demonstrate that each cluster accumulates 7.7 (±4.1) rosettes and, thus, GLOs. Finally, always in Sultan and Heck (2003), the authors show that elements in a cluster are located within 350 μm from the cluster mean location. Taking into account this physiological information, a clustering algorithm capable of generating clusters with a defined dimension is needed. The elements inside the cluster must comply with the distance constraint. Authors developed an algorithm whose goal is to divide several points (inserted in a 3D volume, characterized by spatial coordinates) into N clusters with a fixed, properly set dimension. The number of clusters N is one of the algorithm inputs. Let us assume that the clusters dimension is NUM_POINT_CLUSTER (±DELTA): in this case, NUM_POINT_CLUSTER is set to 8 and DELTA is set to 4 according to Sultan and Heck (2003). In the initialization phase, the algorithm computes the centroids in a pseudo-random way. Once the coordinates are initialized, the algorithm computes the Euclidean distances between the GLOs and the centroids to identify the nearest centroid for each GLO. The algorithm computes the K-nearest centroids for each GLO (in this work, K = 100) and sorts them in ascending order, on the basis of the GLO-centroid distance. At the end, all these data are stored in the *data_clusters* structure, including these fields:

- GLO ID (*ID*_*glo*_);
- Nearest centroid ID (*ID*_*primary*_);
- Distance GLO *ID*_*glo*_ and centroid *ID*_*primary*_;
- Structure that contains ID and distances of the K-nearest centroids of the GLO *ID*_*glo*_.

Each GLO is temporarily assigned to its nearest cluster. At this point, the algorithm computes the dimensions of each cluster (i.e., how many GLOs have that specific centroid as the nearest). If the cluster dimension is out of the range NUM_POINT_CLUSTER (± DELTA), its ID is stored in one of the following arrays:

- *buffer_low*, which contains the IDs of the centroids with a dimension lower than NUM_POINT_CLUSTER – DELTA;
- *buffer_high*, which contains the IDs of the centroids with a dimension higher than NUM_POINT_CLUSTER + DELTA.

The next step aims to reduce the centroids present in the *buffer_high*, so that its dimension can be within the range presented above. A *for* loop removes the *extra* elements in the clusters present in the *buffer_high* array and assigns them to other clusters with lower dimensions. The algorithm tries to select a cluster that has a dimension lower than NUM_POINT_CLUSTER – DELTA and, at the same time, is one of the nearest for the GLO that will be moved. Otherwise, it searches for another cluster in the volume giving priority to the ones with a dimension lower than NUM_POINT_CLUSTER + (DELTA/2).

#### Serial and Parallel Network Simulation

This section illustrates the serial and parallel codes developed to simulate the granular layer activity. In previous works (Florimbi et al., 2016, 2019), authors developed the GOCs and GRCs simulators where the neurons were not connected and their activities were evaluated in parallel. These works validated the GOCs and GRCs behaviors reproduced by the simulators. Moreover, this phase was of crucial importance to evaluate the GPU technology in this kind of applications. The significant speed-up obtained comparing the serial and parallel simulators demonstrates that the GPU is a suitable technology for neuronal simulations. For this reason, authors developed also a CUDA version of the granular layer simulator to exploit single and multi-GPU systems.

Once the *connection matrices* have been generated in the network design step, the GRC and GOC simulators can be integrated to reproduce the activity of the cerebellar granular layer.

In the *Initialization* phase, all the variables related to all the cells and their synapses are declared and initialized in a structure called *grc_cell* for the GRCs, and *goc_cell* for the GOCs. Moreover, each structure contains two further structures (one for the excitatory and one for the inhibitory connections), the membrane potential *V*_*m*_, the synaptic current *I*_*syn*_, the ionic channel current *I*_*ion*_ and conductance *g*_*ion*_, all the gating particles for each ionic channel, and the calcium Nernst potential. Each structure related to the connections contains an array storing the spikes that occur in the synapses. Then, in the *MF signal initialization* phase, the configuration of the simulation protocol, described in section Computational Results, has to be initialized, deciding how the MFs provide inputs to the network. At this point, the algorithm can start evaluating the network activity. For each *t-*th time step, the algorithm evaluates the synaptic and cellular activities of the GOCs and of the GRCs. The *for* loop that iterates over the simulation time is shown in Algorithm 1 at line 4.

**Algorithm 1:** Network simulation.

1 Connection matrices reading;
2 Initialization;
3 MF signal Initialization;
4 **for** t←0 **to** t_end_
5 **for** n←0 **to** n_goc_
6 GOC Synaptic activity computation;
7 GOC Cellular activity computation (solve Equation 2 for each ion);
8 GOC sum currents and conductances;
9 GOC membrane potential update (solve Equation 9);
10 Send signals to granule cells;
11 **end**
12 Gap junctions currents update;
13 **for** n←0 **to** n_grc_
14 GRC Synaptic activity computation;
15 GRC Cellular activity computation (solve Equation 2 for each ion);
16 GRC sum currents and conductances;
17 GRC membrane potential update (solve Equation 9);
18 Send signals to Golgi cells;
19 **end**
20 **end**
21 Write results;
22 **end**

Inside this loop, lines 5 and 13 indicate the loops iterating on the GOCs and GRCs. For the *t*-th time step, the algorithm starts evaluating the GOCs synaptic activity (line 6), by solving the pre-synaptic and post-synaptic terminal models. In particular, the algorithm checks if some inputs have occurred in the GOCs basal and apical dendrites. To model this aspect, three buffers (*spike queues*) have been allocated in each *goc_cell* structure storing the inputs from the basal dendrites, the apical dendrites and the inhibitory synapses. The GOC dendrites are modeled as passive components characterized only by an axial resistance, which causes a delay in the signal transmission. This kind of representation allows analyzing, at each time step, if one or more inputs occurred in the different dendrites of the cell. Algorithm 2 shows this process.

**Algorithm 2:** Synaptic activity computation.

1 **for** *t* ← 1 to *t_*end*_* **do**
2 **for** *i* ← 1 to *n_*goc*_* **do**
3 **for** *j* ← 1 to *buffers* **do**
4 **if** *spike* **then**
5 compute neurotransmitter concentration;
6 remove the spike from the queue;
7 **end**
8 **end**
9 solve the AMPA, NMDA and GABA kinetic schemes;
10 compute the currents that flow in the receptors;
11 *I_*syn*_ = I_*AMPA*_ + I_*NMDA*_ + I_*GABA*_;*
12 …
13 **end**
14 …
15 **end**

The *for* loops that iterate on the time steps and on the GOCs number are in lines 1–2 of Algorithm 2. Then, the algorithm checks if, in each buffer (Algorithm 2, line 3), a spike has occurred in the current time *t* (Algorithm 2, line 4). If the spike occurs, the algorithm solves the three-state kinetic scheme, cited above, to compute the neurotransmitter concentration (line 5). Once the input has been evaluated, the spike is removed from the spike queue. Once all the inputs have been evaluated, the algorithm solves the receptors schemes to compute the currents that flow in the channels present in AMPA, NMDA, and GABA receptors (lines 9–10). Finally, the synaptic current *I*_*syn*_ is updated and the *GOC Synaptic activity computation* phase ends. The flow of Algorithm 1 continues evaluating the GOCs cellular activity (line 7): the value of the gating particles of each ionic channel is updated and, then, the channel conductances and currents are computed. At this point, the current and conductance contributions are summed and included in the membrane potential update. Moreover, in this last phase of the GOCs activity, the gap junctions currents are also considered. In the first iteration (*t* = 0), their value is initialized to zero since all the cells have the same membrane potential. Then, their values will be updated on the basis of the membrane potential difference between the cells linked through gap junctions. The last phase of the GOCs activity (*Send signals to granule cells*) manages the signals exchange between the GOCs and the GRCs. In fact, as said before, the signals travel along the GOCs axons that enter the GLOs, where the GRCs dendrites receive the signals from the GOC. At this point, the algorithm evaluates if the considered GOC generates a spike: if yes, the algorithm searches the GRCs linked to that cell through the connection matrix. Then, it stores the spike time in the suitable GRC spike queue. Here, two considerations are necessary: the first is that the GOC axon is modeled as a passive component. For this reason, the spike time is computed by adding a delay caused by the axonal resistance. The second is about how the GRCs dendrites have been modeled. As said before, the convergence rate between GLOs and GRCs is lower than the GOCs one (Solinas et al., 2010; D'Angelo et al., 2016), thus GRCs have 3–5 dendrites that enter the GLOs. For this reason, the four GRC dendrites have been represented with the same number of buffers for the excitatory and inhibitory connections.

Once all the GOCs signals have been stored in the suitable GRCs buffers, the *for* loop that iterates on the GOCs ends. At this point, the algorithm computed a new membrane potential value for all the GOCs and, then, the gap junctions currents are updated (*Gap junctions current update*). Then, for the same *t*-th iteration, the algorithm continues analyzing the GRCs synaptic and cellular activities. In the *for* loop that iterates on the GRCs, the algorithm starts to evaluate the synaptic activity as shown in Algorithm 2 for the GOCs: the only difference is that, in this case, each GRC has four buffers for the excitatory connections and four for the inhibitory ones. Clearly, the spikes generated by the GOCs in the *t*-th time step are not considered by the GRCs in this iteration. Once the membrane potentials have been updated, the action potentials generated by the GRCs are sent to the GOCs (*Send signals to Golgi cells* phase). In particular, as said before, the GOC receives excitation from the GRC through their ascending axons and/or through their PFs. Once all the GRC have been evaluated, the code can continue with the next time step (*t* + Δ*t*).

**Figure 4:**
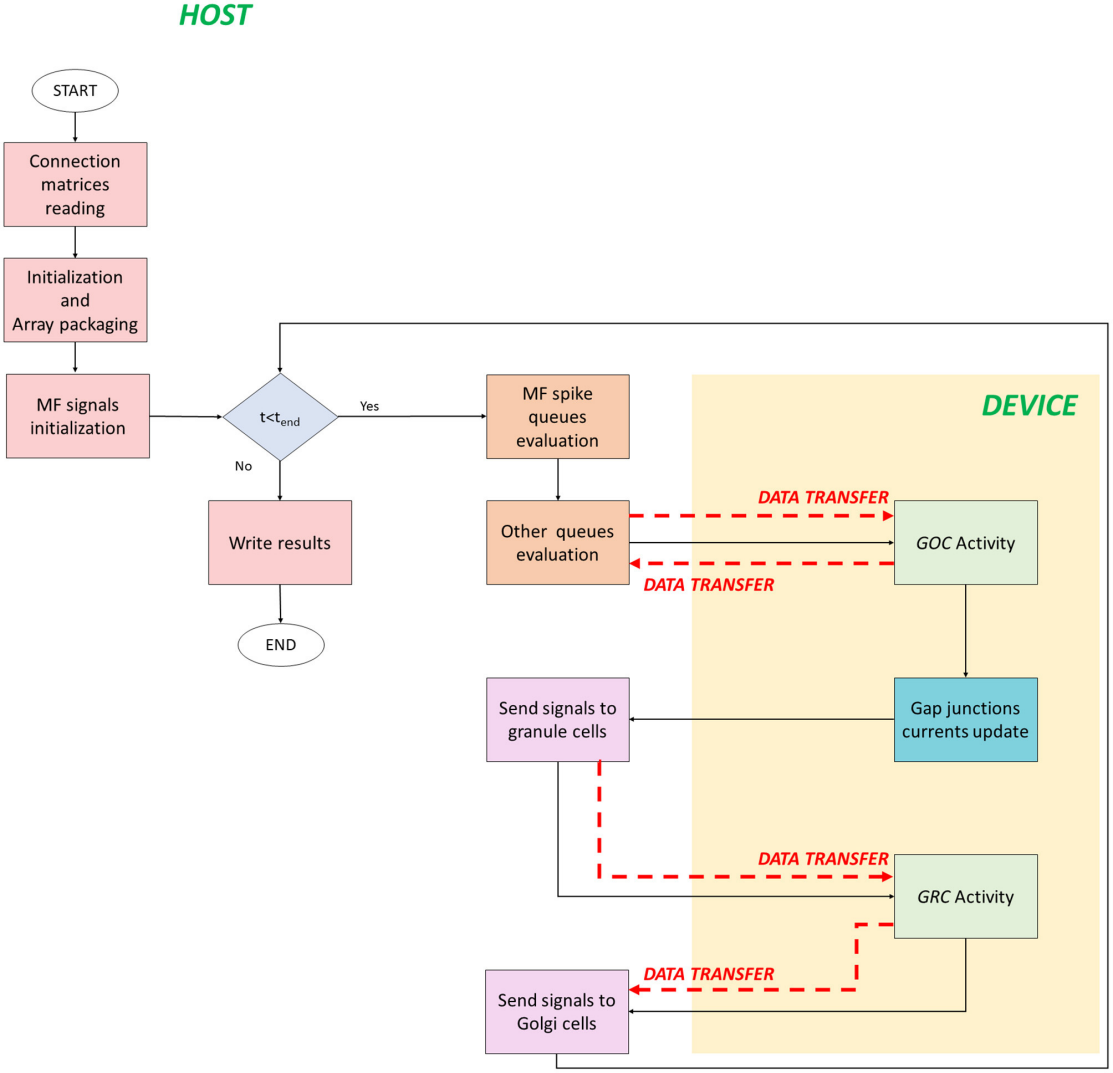
Parallel flow for single-GPU system. The flow starts on the host where the *for* loop iterates on the time steps. The signals exchange is performed on the host, while the neurons activity computation is performed on the device (yellow box). The black arrows indicate the flow, while the red dashed arrows indicate the data transfers between host and device, and vice versa.

The serial version of the granular layer network has been used as a basis for the development of two parallel codes, written in C/CUDA language. Figure 4 shows the flow of the first parallel version, which runs in a single-GPU system.

The code starts on the host where the *connection matrices* are read (*Connection matrices reading*) and the variables are initialized. In addition to the initialization of the variables, in the second phase, the algorithm prepares the data to be transferred from the host to the device memory. This phase is crucial to reach high performance and to reduce the computational times. In fact, if not properly managed, the data transfer could be the bottleneck of the process. In order to prevent this potential slow-down, all the data related to the cells have been stored at contiguous memory addresses, trying to minimize the bus activations during the transfers. The idea is to create a 1D array and to join the data according to their physiological meaning. In this phase (*Initialization and Array packaging*), the data related to the cellular activity and to the initialization of the pre-synaptic and post-synaptic models are prepared. On the other hand, the input signals will be set for the transfer after the configuration of the simulation protocol in the successive phase. In fact, in *MF signals initialization*, a protocol is chosen and the spike queues for each MF are generated. In this case, the authors chose to not transfer all the spikes queues of all the MFs from the host to the GPU global memory in order to not increase the memory usage. Once the protocol is generated, the *for* loop that iterates on the time steps begins. The *MF spike queues evaluation* phase has been introduced to properly manage the data transfers of the MFs queues. Since it is not efficient to transfer all the queues in the global memory, at each time step the algorithm evaluates if the MFs have generated an input signal. If yes, that input is stored in a temporary queue of the cell linked to that MF. Notice that each queue of all the MFs is evaluated in parallel exploiting a multicore strategy with the Application Programming Interface (API) OpenMP. It is a parallel programming model for shared-memory multiprocessors that provides a wide set of directives and strategies for the parallelization of loops and program sections through the *#pragma* directive. This statement is placed before the loop that should be parallelized. Moreover, in this directive, the variables or array are expressed as *private* to a single thread or *shared* among all the threads. In this case, at each current time, the MFs *for* loop is parallelized to simultaneously check the GRCs and GOCs linked to the MFs that have generated a spike. Finally, the algorithm generates two arrays of flags (one for GOCs, *goc_spikeMF* and one for the GRCs, *grc_spikeMF*) that contain the value 1 if the corresponding GOC or GRC received an input from the linked MFs. The flags are equal to zero if the corresponding cell is not stimulated. In this way, at each time step, the algorithm will transfer from the host to the device global memory, only two arrays of dimension *n*_*goc*_ × *sizeof(int)* and *n*_*grc*_ × *sizeof(int)*, instead of all the MFs queues. In the O*ther queues evaluation* phase, other arrays are prepared for the transfer. In this code, the queues/buffers, already defined in the serial code, are present together with their related arrays of flags, exploited to transfer data to the global memory at each time step. For this reason, the GOCs have a buffer and an array of flags for the apical, basal and inhibitory connections (*goc_spikeAPIC, goc_spikeMF*, and *goc_spikeINH*, respectively). The GRCs have two flag arrays: one for the excitatory and one for the inhibitory connections. These two arrays are properly managed to transfer the signals stored in the four excitatory and inhibitory buffers present in the host. On the device global memory, space has been allocated to store the synaptic buffers and the gap junctions currents, i.e., an array whose dimension is *n*_*goc*_ × *n*_*gap*_ × *sizeof(float)*, where *n*_*gap*_ is the number of connections through gap junctions for each cell. All the *flag* arrays are transferred to the GPU global memory (red dashed arrows in Figure 4). At this point, the activity of all the GOCs, at the *t*-th time step, can be evaluated simultaneously on the device. This part (*GOC Activity* phase) represents a *kernel*, thus a function performed by parallel threads. For this reason, the device generates a number of threads equal to the number of GOCs, so that each thread can compute the activity of a specific GOC. Threads are organized in *blocks* that, in turn, constitute a *grid*. The block dimension (i.e., how many threads a block contains) is set as multiple of 32, according to the *warp* definition, to optimize the scheduling carried out by the NVIDIA Giga Thread scheduler (NVIDIA, 2019).

Before the kernel invocation, the code computes the grid dimension (i.e., the blocks number) as [*n*_*goc*_*/n*_*thread*_*block*_], where *n*_*goc*_ is the GOCs number (i.e., the total number of threads needed), and *n*_*thread*_*block*_ is the number of threads in a block, set to 32. If the remainder of the division is not equal to zero, the grid dimension is incremented by one. In this case, the last block contains more threads than necessary: this turns out in inactive threads assigned to the last block. Despite this, the inactive threads cannot be avoided because each block must have the same number of threads. Once all these parameters have been defined, the kernel can be activated. The first step of this phase is the data transfer from the global to the local memory of the device. All the threads within a block can access the same portion of the local memory and, for this reason, the goal is to copy the parts of the arrays that are needed by the threads in the block in this memory. In this way, the memory access latency diminishes. On the other hand, the local memory has a reduced size (dozens of KB) and, for this reason, not all the data can be transferred. In this way, only the arrays and variables that are the most used in the kernel are stored in the local memory. Moreover, these variables are accessed multiple times by all the threads in the block. When this first set of memory transfers has been concluded, all the threads start evaluating in parallel the GOCs activity. The evaluated phases are those shown for the serial code and represented in Algorithm 1 lines 6–9. Once the GOCs activity is computed, all the updated data (i.e., membrane potential, kinetic scheme variables, gating particles, Nernst potentials, calcium concentration, and so on) are stored in the global memory because they will be used in the next iteration. Once this kernel has finished, another one starts computing the gap junctions currents. This kernel has the same number of threads and blocks previously defined since each thread evaluates the gap junctions connections of each GOC. The currents are stored in the device global memory and evaluated in the next GOCs activity evaluation. Once the kernels finished, two arrays are transferred from the device to host: the former stores the membrane potential of all the GOCs; the latter, called *flag_golgi_spike*, has size *n*_*goc*_ and, for each cell, stores a flag whose value is 1 if the corresponding GOC has generated a spike. The first arrays is then used to record the potentials in the mass memory of the system. As described above, this spike travels along the GOC axon, which enters in the GLOs, where the GRCs dendrites are hosted. At this point, exploiting the *connections matrices*, the code evaluates on the host which are the GRCs linked to the GOCs that have generated a spike. For these GRCs, the spike time is computed and stored in their buffers for the inhibitory connections, since the GOCs provide an inhibition. In this way, all the GRCs inhibitory buffers are updated and, then, analyzed in order to see if they contain inputs that have to be evaluated by the GRCs in the current time *t*. This means that the flags of the array *grc_spikeINH* assume the value 1 if the corresponding GRC received an inhibitory signal to evaluate in *t*. Clearly, also in this case, the GRCs do not evaluate the GOCs spikes generated at the same time iteration. The flag array is then transferred from host to device, where a new kernel is invocated (*GRC activity* in Figure 4) to evaluate the activity of all the GRCs, for the *t*-th time step, simultaneously. In fact, the device generates a number of threads equal to the GRCs number. As for the GOCs activity, also in this case parts of data are transferred from the global to the local memory. Then, each thread computes the activity of one GRC, performing all the functions indicated in Algorithm 1 lines 14–17. At the end, all the updated variables are transferred back from the local to the global memory, in order to be used in the next time iteration. For the GRCs, two arrays are also transferred from the device to the host: the first stores the neurons membrane potentials, and the second (*flag_grc_spike*) stores flags that indicate if the corresponding GRC has generated a spike. At this point, the code is processed on the host, where the array *flag_grc_spike* is analyzed. In this case, the code checks which are the GOCs linked to the GRCs (that have generated a spike) through the ascending axon and the PFs. For all these GOCs, the spike time is computed and stored in the apical connections buffer, which is then evaluated to update the *goc_spikeAPIC* array. The flags values will be set to 1 if the corresponding GOC is linked to a GRC that has sent a signal. The *Send signals to Golgi cells* phase is the last and the code can proceed with the next iteration.

**Figure 5:**
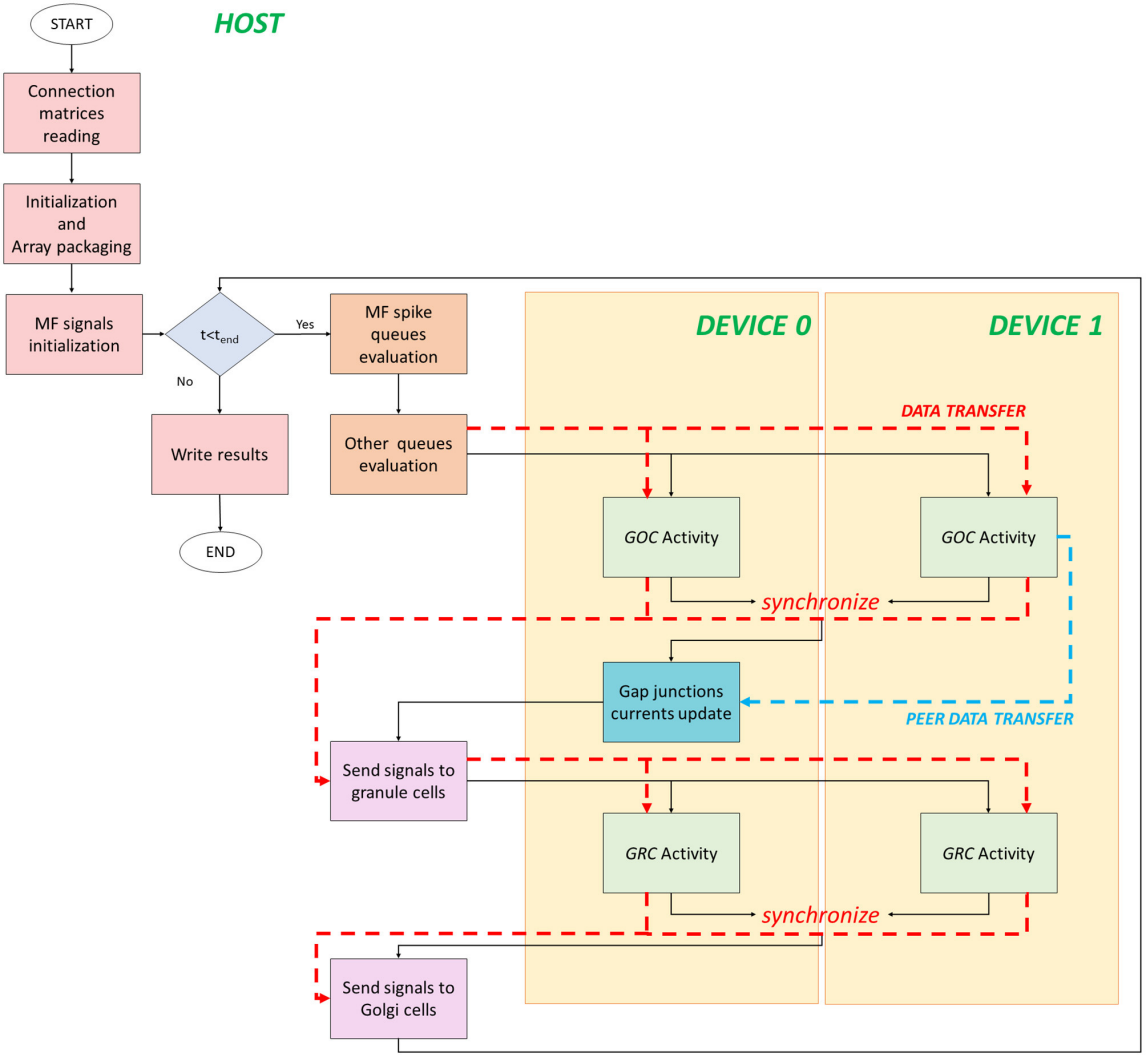
Flow parallel version for the multi-GPUs system. The flow starts on the host, where the variables are initialized, and data are prepared for the transfers to the global memory of the devices. The GOC Activity and GRC Activity is managed by the two devices. The black lines indicate the flow, the red dashed lines indicated the host–device (and vice versa) transfers, and the blue dashed line indicates the transfer between devices.

The flow of the multi-GPU parallel version is shown in Figure 5. It starts on the host where data are initialized and prepared for the transfer. Despite the previous version, data have to be transferred from the host to two different devices. In fact, the neuronal activity evaluation is split between the two boards and, in particular, each one processes the activity of half of the neurons. Therefore, data related to the first half part of the cells are transferred to the *device 0* global memory, the others to the *device 1* one. For each simulation time step, the queues evaluation is the same done in the single-GPU version. The only difference is how data are prepared and split to be transferred to two devices. In order to invocate the *GOC Activity* kernel on two boards, firstly the kernel parameters have to be set. The number of threads in each kernel is given by (*n*_*goc*_*/2)/n*_*thread*_*block*_, where *n*_*thread*_*block*_is always set to 32. If the number of cells is not an even number, the threads number in one of the two devices is incremented by one. In order to perform two kernels simultaneously, CUDA provides the *streams* to concurrently execute and overlap kernels and data transfers (Rennich and NVIDIA, 2014). The streams carry out the transfers (indicated with red dashed lines in Figure 5) and activate the kernels on both the devices. All the previous considerations related to the transfers from the global to the local memory can be also done in this case for each board. Once the *GOC Activity* of all the GOCs is evaluated, the kernels end, and the flow is synchronized. At this point, the arrays that store the GOCs membrane potentials and flags (which indicate if each a GOC has generated a spike or not) are transferred from the device to the host memory. Moreover, a data transfer between the two boards is performed (light blue dashed line in Figure 5). In fact, the kernel related to the gap junctions currents computation is entirely performed on the device 0 since the code needs the membrane potential values of all the GOCs to update these currents. Indeed, each GOC is connected through gap junctions to other GOC which might not be located on the same device. For this reason, the membrane potentials evaluated by the device 1 are transferred to the device 0 through a *cudaMemcpyPeerAsync* function, which allows to directly transfer data between GPUs on the same PCI Express bus bypassing the CPU host memory.^1^ Once the gap junctions currents have been updated and stored in the device 0 global memory, the flow returns on the host where the signals are exchanged and the flags arrays updated, as explained before. Then, the arrays are transferred on the two boards in order to activate the *GRC Activity* kernel on the two devices. As for the GOCs, each kernel evaluates on each board the activity of half part of the GRCs. Once the flow is synchronized, the arrays containing the GRCs membrane potentials and the flags, indicating the spike presence, are transferred from the devices to the host. At the end, the signals are exchanged between GRCs and GOCs and stored in the GOCs buffers for the apical connections. At this point, the flow can continue with the next iteration.

#### Graphical User Interface

**Figure 6:**
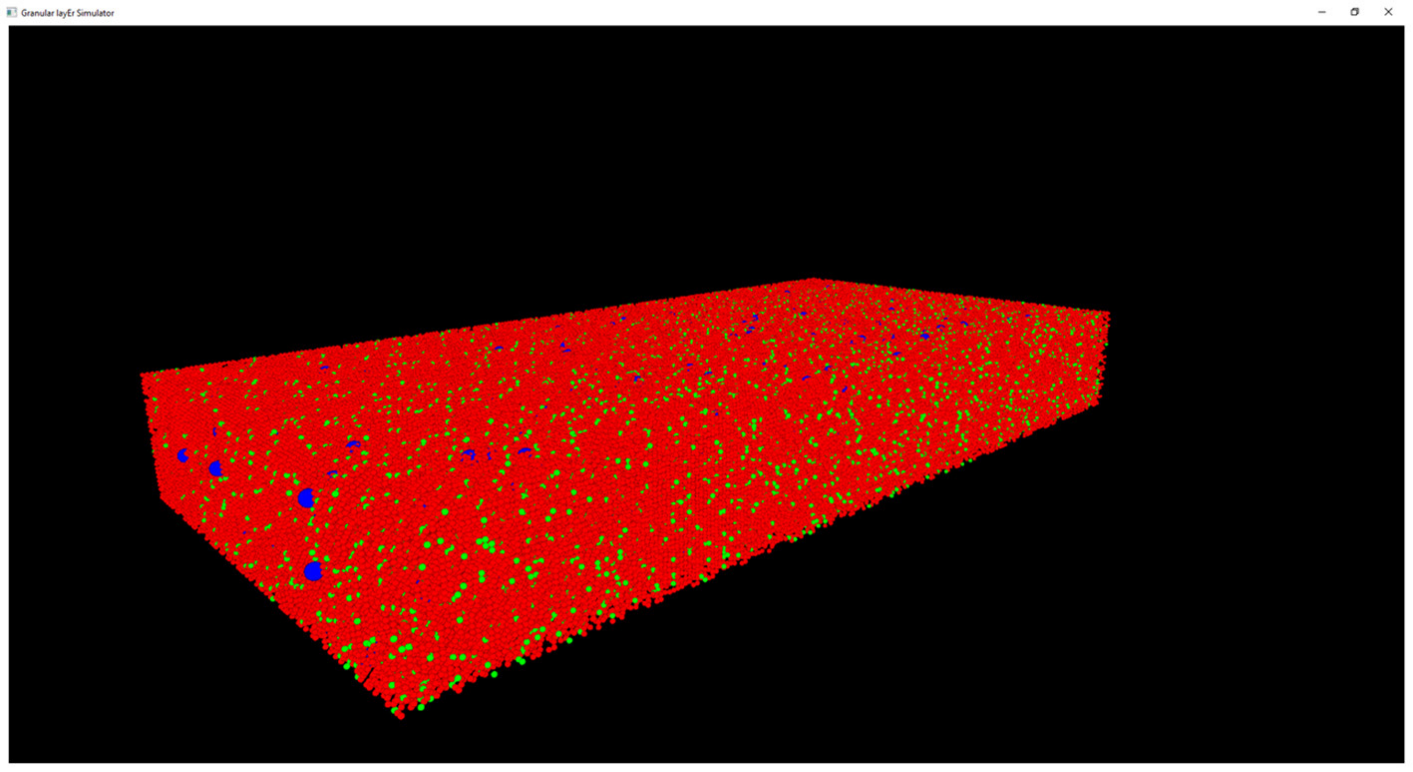
Complete view of the network. Main panel where the complete network (with dimension 600 × 150 × 1,200 μm^3^) is shown. Only the GRCs (red) and GOCs (blue) soma have been displayed. The GLOs have been represented as green spheres.

**Figure 7:**
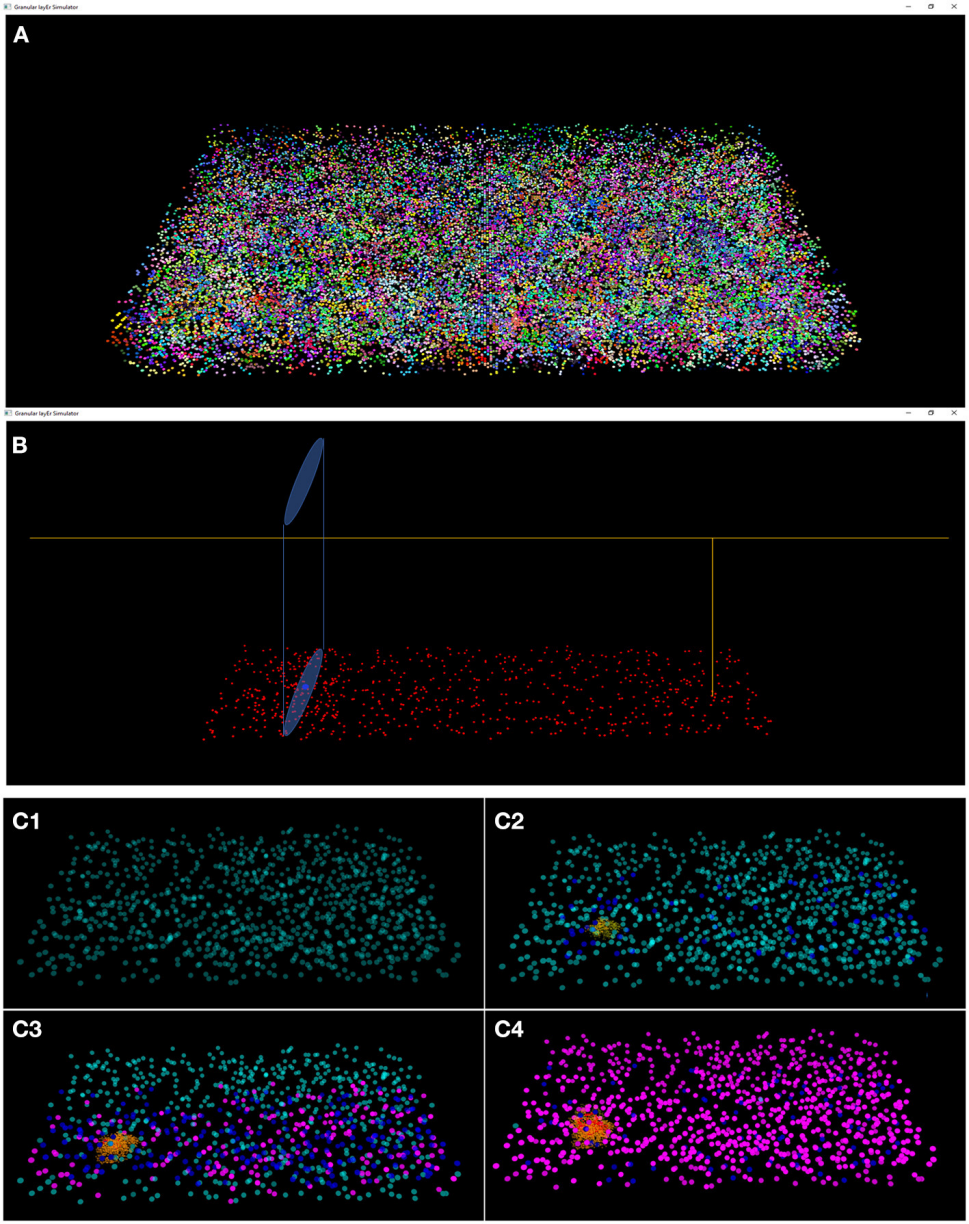
Three tasks of the network. **(A)** MFs branch in the cerebellum forming clusters of GLOs. All the GLOs that belong to a cluster are shown in the same color; **(B)** example of connection between the GRCs (red) and the GOC (blue) through PFs: the GRC ascending axon branches in PF (yellow) that crosses the space dedicated to the GOC apical dendrites (light blue cylinder); **(C)** Four frames of the center-surround organization: **(C1)** only the GOCs have already generated a spontaneous spike; **(C2)** some GRCs and GOCs are stimulated by the active MFs; **(C3)** the core of the center-surround organization is more visible. The GOCs connected through PFs are more excited than the others; **(C4)** final frame of the center-surround.

The results of the design and of the network neuronal simulations can be graphically and quantitatively analyzed through a graphical interface, developed with the OpenGL API in order to achieve a GPU-accelerated rendering (Sellers et al., 2015). The main task of this graphical interface is to display the elements in a 3D volume, considering the spatial coordinates computed in the network design stage. In Figure 6, the main panel shows the granular layer network, with the dimension considered in this work (i.e., 600 × 150 × 1,200 μm^3^). In particular, the GLOs are represented in green, the GRCs in red and the GOCs in blue. The interface is also useful to analyse the connections between GLOs and neurons, the MFs clustering and to watch the simulation output. As an example, two tasks have been shown in Figure 7. In particular, Figure 7A shows how the MFs branch in the cerebellum forming clusters of GLOs, represented with different colors. Figure 7B shows an example of connections between GRCs and GOCs through PFs. All the GRCs in red are the ones selected by the algorithm following the rules presented in section Network Design. Only the ascending axon and the PF of one GRC are represented to show how the PF crosses the space dedicated to the GOC apical dendrites, generating a connection. As far as the simulation tasks are concerned, the algorithm evaluates the membrane potential of each element in all the time steps of the simulation. In each time step, it changes the color of the elements that are generating a spike. In this way, the user can graphically analyse how neurons react to particular stimuli. In the center-surround simulation (Figure 7C), which will be described in section Computational Results, a particular technique is adopted to show the spiking neurons: at the beginning, all the neurons are not visible in the volume. As soon as a cell generates a spike, it will be shown with a color whose tone becomes darker as the spikes number increases.

## Results and Discussion

### Neuron Placement and Connection Analysis

**Figure 8:**
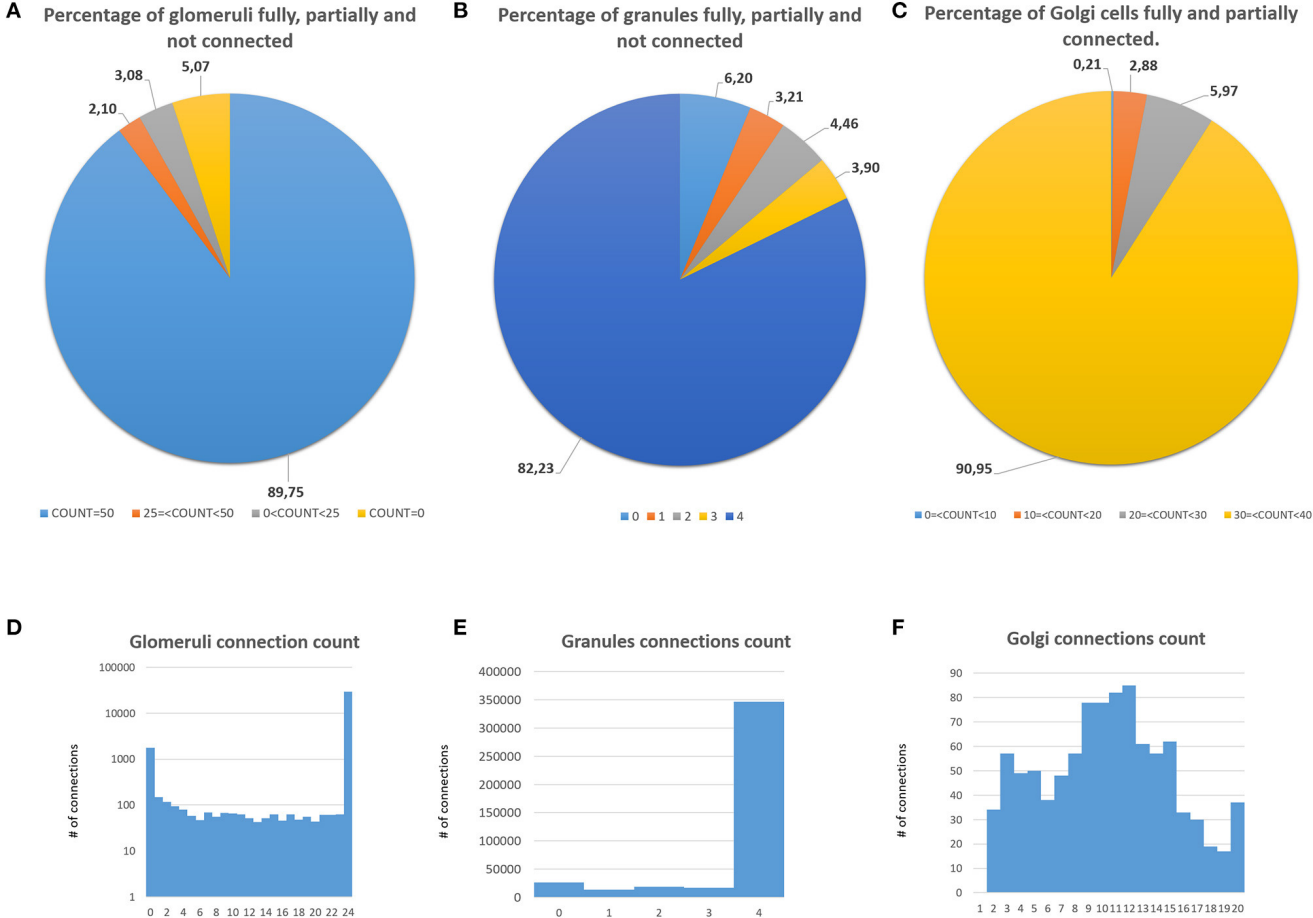
Percentage of connection and connections count of GLOs, GRCs, and GOCs. **(A)** Percentage of GLOs fully (COUNT = 50), partially (25 ≤ COUNT < 50, 0 ≤ COUNT < 25) and not linked (COUNT = 0) to the GRCs; **(B)** percentage of independent connections between GRCs and MFs (through GLOs). Each GRC can be linked to four different GLOs at most; **(C)** percentage of GOCs basal dendrites linked to the MFs (through GLOs). **(D)** Connections count of GLOs. **(E)** Connections count of GRCs. **(F)** Connections count of GOCs.

**Figure 9:**
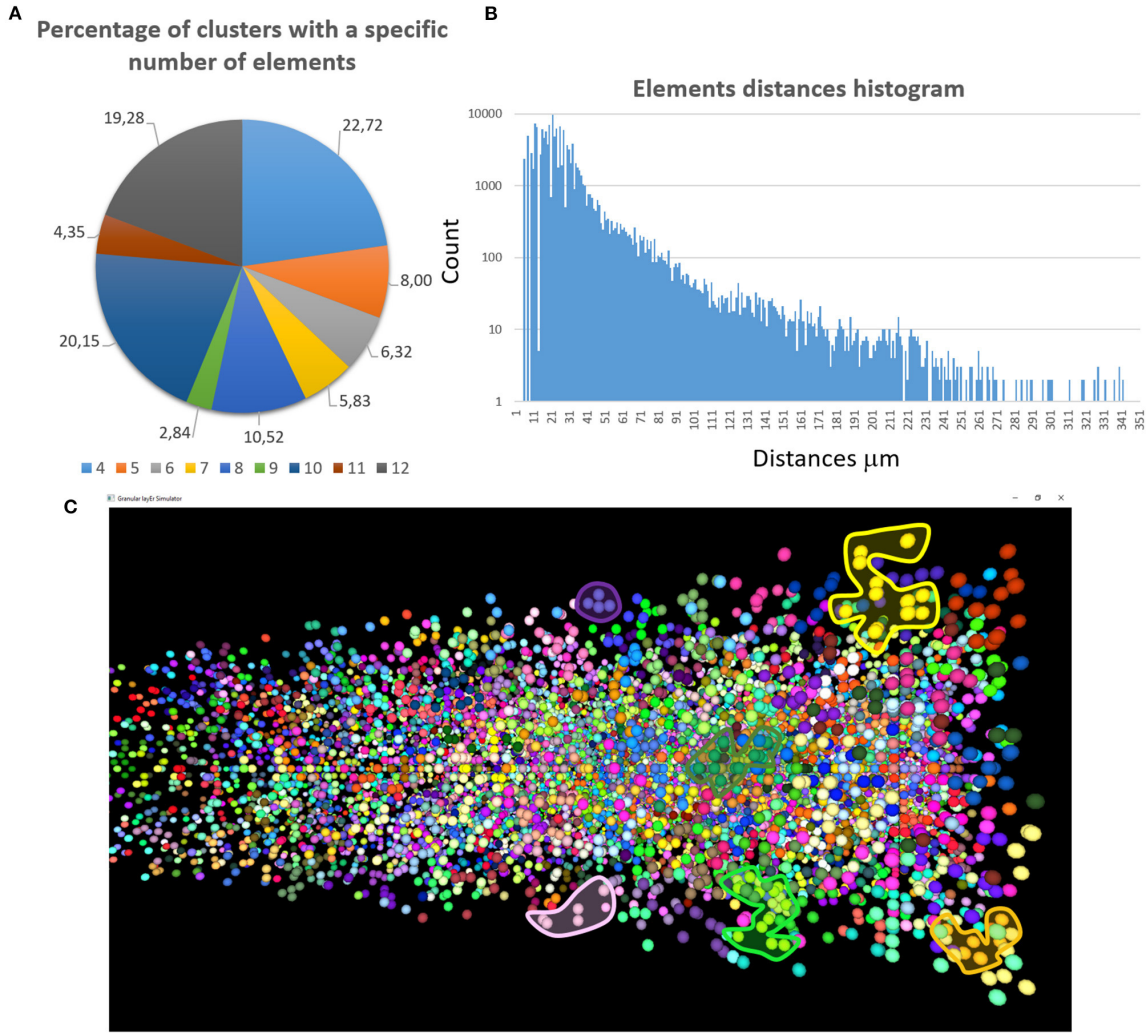
MFs clustering. **(A)** The graph shows the percentage of clusters with a different number of GLOs (4 ÷ 12); **(B)** the graph shows the distances distribution; **(C)** the GLOs within the same cluster are displayed in the same color. By way of example some clusters have been highlighted.

The first validation concerns the evaluation of how many elements can be correctly placed by the proposed network design algorithm. The analysis presented below is performed after running the design algorithm 20 times with different random seeds. Authors find that the algorithm always places the 100% of GOCs and GLOs, and 98% of the GRCs. This amount of not placed cells is negligible in terms of the correct evaluation of the network activity. Moreover, authors carefully analyzed the *connection matrices* to evaluate the percentage of links established during the network design step. The analysis of the *link_grc_glo* matrix reveals that the algorithm completely fills the 50 available places of the 89.75% of the GLOs (Figure 8A). In only the 5.07% of cases, the GLOs are not linked to the GRCs dendrites and, in the other cases, the number of dendrites that reach the GLOs is between 1 and 49. Moreover, the evaluation of this connection matrix highlights that 82.30% of the GRCs sends its dendrites to four different GLOs, satisfying the convergence constraint (Solinas et al., 2010; D'Angelo et al., 2016) (Figure 8B). Moreover, this means that this percentage of GRCs is excited by four different MFs. In other cases, the GRCs are partially linked to 3 (3.90%), 2 (4.46%), and 1 (3.21%) GLOs/GLO. Only in the 6.20% of the cases, the GRCs are not connected to GLOs. The fact that not all the GLOs and GRCs are entirely linked is not a limit of the algorithm that is based on convergence/divergence average values (or ranges) taken from the literature. For this reason, performing the connections not reaching the maximum number of the expected elements is not an error. Instead, it provides more variability and realism to the network. These considerations are also valid concerning the connections presented below. Figure 8C shows the results of the analysis conducted on the *link_gocdb_glo* matrix, containing the GLOs where the GOCs spread their basal dendrites. The algorithm is capable of fully connecting the 90.95% of GOCs to 40 MFs. In the 9.06% of the cases, the GOCs are linked to <40 GLOs, but all the GOCs receive at least the signal from one MF. Figures 8D–F also show the count of connection for GLOs, GRCs and GOCs. Considering the inhibition that the GRCs receive from the GOCs through their axon, the matrix *link_goca_glo* evaluation highlights that the developed function generates the 94.97% of these connections. Moreover, considering the GRCs–GOCs connection through ascending axon and PFs, the algorithm fully connects the GOCs to the GRCs, following the convergence/divergence rates presented in section Overview of the Cerebellar Granular Layer Model. Finally, the gap junctions connections evaluation highlights that the 96.60% of GOCs are connected to two other cells (Vervaeke et al., 2010), while only the 1.85% shows one link and the 1.54% is not connected since those cells are located in the borders of the volume. The algorithm that reproduces the MFs branching creates all the clusters with a number of GLOs in the range of 7.7 (±4.1), satisfying the constraint proposed in Sultan and Heck (2003). The number of MFs present in this network configuration is 4051. Figure 9A presents a graph showing the percentages of clusters with a different number of GLOs. It is possible to notice that the algorithm creates the 22.72% of clusters with 4 elements, 20.15% with 10 and 19.28% with 12. Moreover, the distances between the elements within the clusters satisfy the constraint of 350 μm. Therefore, the procedure described in section Network Design does not alter the distances distribution inside a cluster. The distances distribution is shown in Figure 9B. Figure 9C shows all the GLOs belonging to a cluster in the same color.

### Computational Results

Simulations have been carried out on a system equipped with an Intel i9-9900X CPU, working at 3.50 GHz, and with 128 GB of DDR4 RAM memory. The system is also equipped with two NVIDIA RTX 2080 GPU (Turing architecture), each one with 2944 CUDA cores, 8 GB of DDR6 memory and working at 1.8 GHz. The boards are connected to the host through a PCI Express 3.0.

The simulations have been also carried out on a single node of an EOS cluster hosted at our University. The node is equipped with two NVIDIA Tesla V100 GPU (Volta architecture), each one with 5120 CUDA cores, 32 GB of HBM2 RAM memory, and working at 1.38 GHz. Each node has an Intel Xeon Silver 4110 CPU, working at 2.1 GHz. Considering the network design stage, the developed algorithm places and connects all the elements in only 235 s on an Intel i9 CPU. In particular, the elements placement takes 31.84 s, while their connections and the matrices generation take 203.16 s.

Considering the layer activity simulation, the differential equations in the neurons models have been solved adopting a first-order Euler method, with a time step equals to 0.025 ms. During the single-cells simulators development, the authors performed several tests to set the optimal time step in order to validate the results against the ones produced by the NEURON simulator (Florimbi et al., 2016, 2019).

To evaluate different neuronal behaviors of the network, several protocols have been developed. Table 2 shows their characteristics.

**Table 2:** Protocols details.

**Protocol ID**	**Background (Hz)**	**TI_**back**_ (s)**	**TF_**back**_ (s)**	**Burst (Hz)**	**TI_**burst**_ (s)**	**TF_**burst**_ (s)**	**#MF burst**
Prot1	1	0 + delta	T_end_	–	–	–	–
Prot2	–	–	–	100	T_rand_	T_rand_ + 0.05	10%
Prot3	1	0 + delta	T_end_	100	T_rand_	T_rand_ + 0.05	100%
Prot4	1	0 + delta	T_end_	100	T_rand_	T_rand_ + 0.05	1%

*The table shows the protocol ID, the background frequency, the instant times when the background starts and ends. Moreover, it shows the burst frequency, the instant times when the burst starts and ends, and the number of MFs activated with the bursts*.

**Figure 10:**
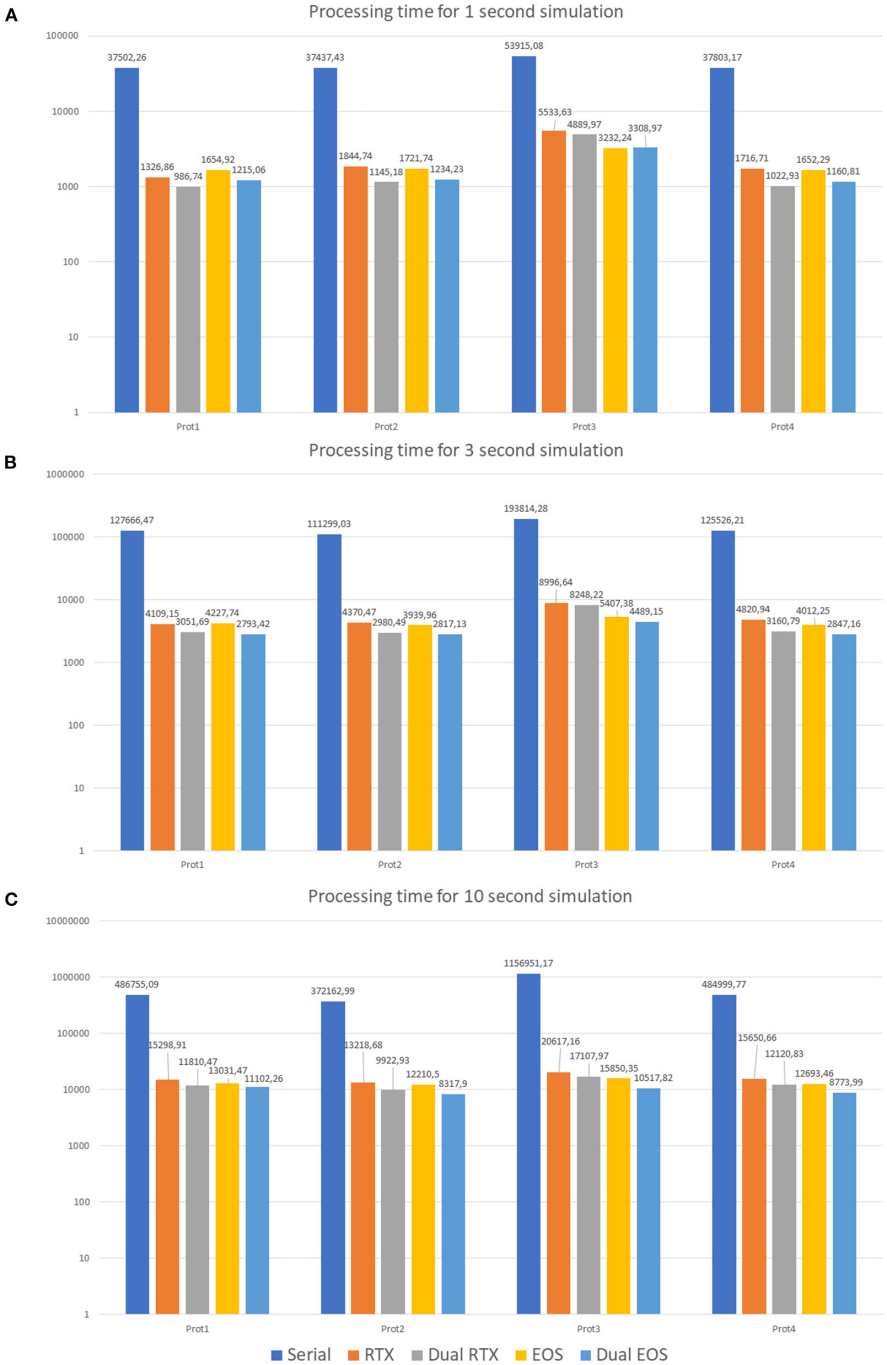
Processing times. 1 s **(A)**, 3 s **(B)**, and 10 s **(C)** neuronal activity simulations on the different test systems. The serial simulation ran on Intel i9 CPU, RTX and Dual RTX refer to the NVIDIA RTX 2080 boards, and EOS and Dual EOS refer to the NVIDIA V100 boards. The graphs show the results of the simulations where the four protocols have been tested (Prot1, Prot2, Prot3, and Prot4). The graphs are in logarithmic scale. The legend refers the three graphs.

**Figure 11:**
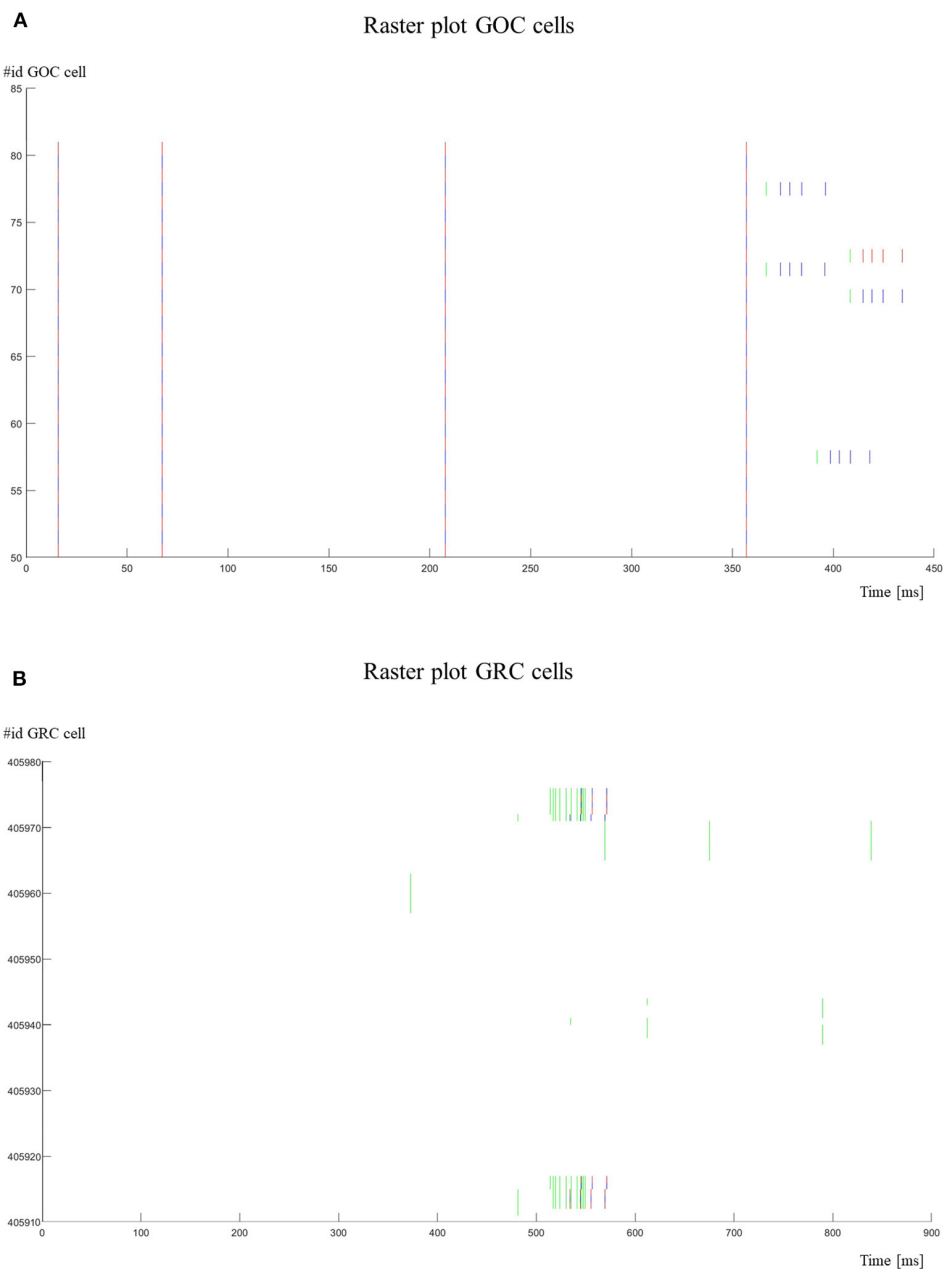
Raster plots. **(A)** The activity of the GOCs (id 50–80) is shown. The cells show a spontaneous firing until they are stimulated (green lines) by MFs. In these cases, their firing frequency is increased; **(B)** the activity of the GRCs (id 405911–405976) is shown. Some GRCs are stimulated with bursts by MFs. It is possible to notice that GRCs generate a spike only after 3–4 stimuli. The red lines refer to the cells with an even id, while the blue lines refer to the cells with an odd id.

The first protocol (*Prot1*) aims at evaluating the network response to a background signal of 1 Hz over all the MFs. These inputs start after a *delta* of 350 ms from the beginning of the simulation and last for the whole activity time (*T*_*end*_). On the other hand, to evaluate the network behavior in response to bursts, 10% of the MFs are activated (*Prot2*). They generate bursts lasting 50 ms and whose initial time is randomly selected. Their frequency is 100 Hz. *Prot3* combines background and bursts in only one protocol. In particular, each MF presents a background stimulus and a burst. This scenario is not realistic from the physiological point of view since it is very improbable that all the fibers are characterized by both these stimuli in these kinds of simulations. However, this protocol has been introduced as a *stress test* to analyse the performance with a huge computational load. Finally, *Prot4* represents a realistic version of *Prot3*. In fact, all the MFs are characterized by a background stimulus but only 1% of them generates a burst during the simulation. Each protocol has been used as input of three different simulations, where 1, 3, and 10 s of neuronal activity have been evaluated. **Figure 11** shows three graphs presenting the processing time (in logarithmic scale) of each simulation on the different test systems. In particular, the *Serial* version has been processed on the Intel i9 CPU. The single and multi-GPU versions have been processed exploiting the NVIDIA RTX 2080 GPUs, and the NVIDIA V100 GPUs. Similarly, Figures 10A–C present the processing times for 1, 3, and 10 s of activity reproduction, respectively. All the elaborations refer to a network with size 600 × 150 × 1,200 μm^3^, hosting a number of neurons equal to ~423,066 and of 32,400 GLOs. This network dimension has been chosen to consider a relevant number of elements and to reproduce the characteristic network behaviors. When analyzing the graphs in Figure 11, we can firstly observe that, as expected, *Prot3* is the slowest among the four tests. In fact, this protocol provides to the network a huge number of inputs, which increases the times that the algorithm has to evaluate the presynaptic model. Taking into account the number of stimuli that are introduced in the network, it can be concluded that the computational time of the serial versions strongly depends on the number of inputs of the protocol. Both in the 1, 3, and 10 s simulations, the highest number of inputs is provided by the *Prot3* (~24,300, ~32,400, and ~60,750 signals on the whole network, respectively), followed by the *Prot4* (~4,250, ~12,350, and ~40,700, respectively), the *Prot1* (~4,050, ~12,150, and ~40,500, respectively), and the *Prot2* (always ~2025). As can be seen from these data, in *Prot1* and *Prot4* the number of inputs is similar, and this small difference does not guarantee that *Prot 4* is always the fastest solution among the two. In these serial simulations, *Prot2* takes always the lowest computational time, as expected. The considerations made on the link between the number of inputs and the processing time cannot be repeated for the parallel versions since different aspects have to be introduced. For example, the data transfers and the access to the device global memory can introduce delays that increase the processing time. Comparing the parallel and serial versions, all the parallel elaborations perform better than the serial ones. In fact, the serial versions of the 1, 3, and 10 s simulations last from 10 to 14 h, from 30 to 35 h, and from 103 to 321 h (i.e., from 4 to 13 days), respectively. These processing times are strongly decreased considering that, in the worst case, the parallel simulation takes about 6 h (considering the 10 s simulation, with one RTX, *Prot3*). Comparing the single-GPU versions (*RTX* and *EOS* in Figure 10), it can be noticed that there are no substantial differences between the processing times, considering the three simulations and the four protocols. This is due to the fact that, even if the EOS GPU (i.e., NVIDIA V100) is equipped with a higher number of CUDA cores than the NVIDIA RTX, this last one features a higher working frequency and a more recent architecture. These characteristics make the difference between the processing times negligible as expected. All the single-GPU parallel versions provide a speedup compared to the serial code processing time. For example, considering the 10 s simulation of *Prot4*, the serial code takes 484999.77 s (i.e., ~5.61 days), to elaborate the network activity. The parallel code on one RTX 2080 GPU takes 15650.66 s (~4.34 h) while the one on the V100 GPU (EOS) takes 12693.46 s (~3.52 h), obtaining a speedup of ~31× and ~38×, respectively. Also in the *stress test* case (*Prot3*), the single-GPU parallel versions perform better than the serial code, providing a speedup up to ~72×. The multi-GPU versions always improve the performances compared to the corresponding single-GPU code. For example, always considering the 10 s simulation of *Prot4*, the processing time is 12120.83 s (~3.37 h) considering two RTX 2080, and 8773.99 s (~2.44 h) considering the two boards in the EOS system. In these cases, the speedup compared to the serial version increases to ~40× and ~55×, respectively. It is worth noticing that the usage of a dual-GPU system does not halve the processing time. This is because some elaborations are performed on a single GPU, moreover, the initialization and the results writing are performed in serial. Finally, not all the memory transfers from the host to the devices can be perfectly overlapped.

Authors also analyzed the code in order to highlight the computational weight of each part of the main *for* loop. The code profiling highlights that about the 95% of the time is taken by the CUDA kernels, the memory transfer account for the 4.6% and only the 0.4% is taken by the host functions. Therefore, there is no reason to implement the spike propagation on GPU. Moreover, the spike propagation could degrade the GPU performance since some parts are strictly sequential.

These results demonstrate that this kind of technology, together with an efficient code development, allows reducing the serial processing times. In this respect, authors decided to perform a very long simulation (50 s) of the neuronal activity using the GES system adopting the *Prot4*. The choice of this protocol has been made since it is the most realistic one, combining the background signals with the bursts. The simulation has been run on the EOS cluster exploiting two NVIDIA V100 GPUs. To reproduce 50 s of neuronal activity, the system takes 49839.68 s (~13 h). This result demonstrates that this system is suitable to reproduce very long neuronal activity, giving the opportunity to study particular behaviors that are not reproducible with other kinds of simulators due to their slower processing times. The GPU technology, together with the optimization developed to efficiently perform the data transfers and the memory accesses and to process the neuronal activity, constitutes an appropriate solution for the network simulation. In particular, this system is capable of fast reproducing a considerable portion of the granular layer, characterized by a high number of neurons, described by complex mathematical models.

Figure 11 shows the raster plots to graphically visualize the network activity in response to *Prot2*. In Figure 11A, it is possible to evaluate the GOCs activity. In particular, these cells generate spontaneous firing and, when stimulated by MFs, they increase their firing frequency. Only some cells are shown (id 50–80) and in a reduced time-window (0–450 ms). On the other hand, the GRCs do not show spontaneous firing and they generate spikes only when stimulated (Figure 11B). In fact, as it is possible to notice from the raster plot, the cell generates a spike after receiving 3–4 stimuli by the MFs.

**Figure 12:**
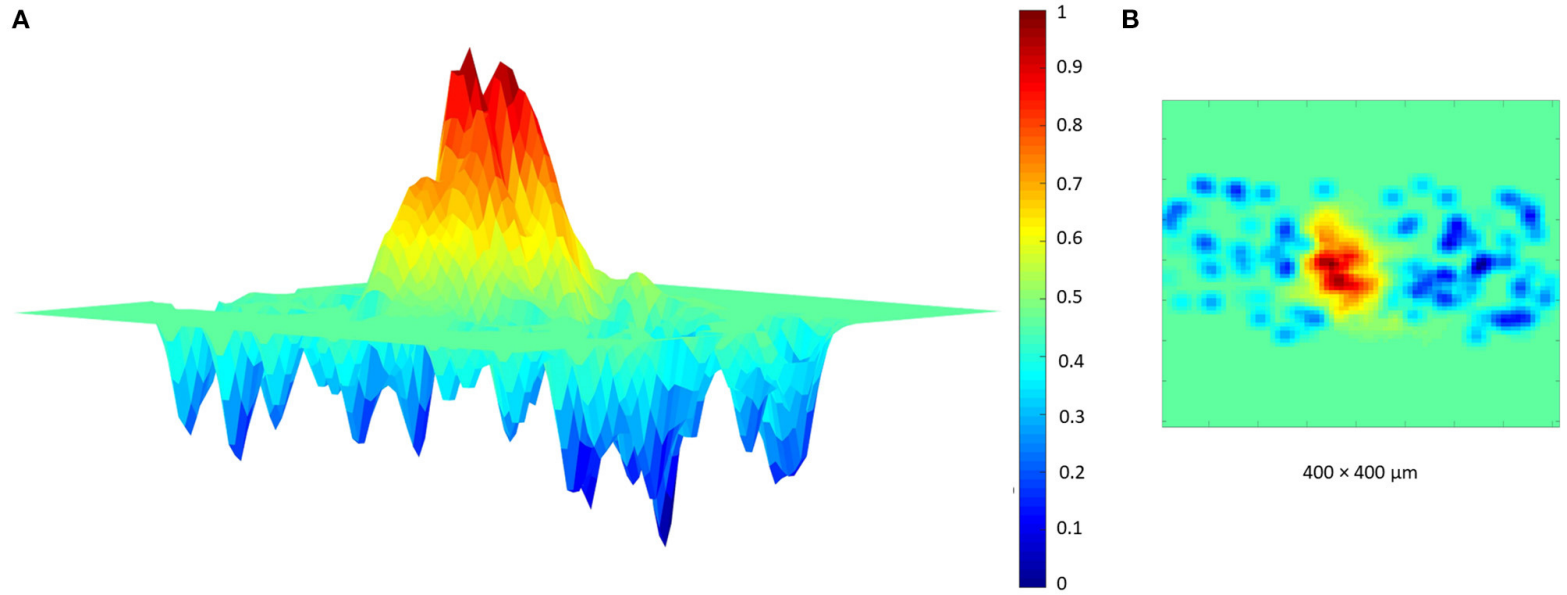
Center surround organization. The MFs stimulate the GLOs with a burst of 50 ms and 150 Hz. The network response is characterized by an excited core caused by the GRC firing (red area). This center is surrounded by an area, where the GRCs response is inhibited by the GOCs. **(A)** Center-surround lateral view. **(B)** Center-surround top view.

Finally, a further validation of the proposed network has been achieved reproducing the typical center-surround organization of the granular layer (Mapelli and D'Angelo, 2007; Solinas et al., 2010; Gandolfi et al., 2014). In fact, several electrophysiological experiments (Mapelli and D'Angelo, 2007; Mapelli et al., 2010a,b) showed that a MFs bundle can stimulate a specific area of the granular layer, generating a central area of excitation and a surrounding one of inhibition. To reproduce this organization, the protocol adopted as input is characterized by the activation of the MFs present in a selected area whose diameter is 50 μm. It is important to highlight that the GLOs, and thus the GRCs and GOCs, excited by these MFs can be also outside this area. In fact, as explained before, each MF stimulates all the GLOs within a cluster and, even if all the elements within a cluster are not so far, it could be possible that they are outside the selected area. Also in this case, it is possible to correctly reproduce the center-surround organization using as input the branched MFs. In particular, in this simulation, the MFs within the selected area are 9 and each one stimulates the GLOs with a burst of 50 ms and a frequency of 150 Hz. Moreover, the entire network is considered (i.e., all the elements can react to an eventual stimulus) and all the connections are switched-on while, in the reference papers, only the area of interest is switched-on. The response of the center-surround shown in Figure 12 is the result of a single simulation run. The burst stimulation causes a central area with a stronger excitation (red area) than the surrounding one (blue area), where the GOCs inhibition limits the rate of GRCs output, overcoming the excitation around the core.

### Memory Occupancy

One of the most important aspects of the simulator is that it is parametric. The user can vary several parameters, such as the volume of the network, to simulate different granular layer configurations. This characteristic makes the system very flexible for what concerns the network construction. Concerning the network design stage, running on the CPU, the code allocates 600 B for parameters used in the network construction and elements connections, whose number is not proportional to the number of neurons or elements. Moreover, the code allocates a memory space proportional to the number of GOCs, GRCs, GLOs and MFs, as shown in Equation (15):

(15) $$\begin{array}{l} MEMdesign=600+15432\;n_{goc}+\;32\;n_{grc}+1292\;n_{glo}\\ \;+296\;n_{MF} \end{array}$$

where *MEMdesign* is the amount of memory expressed in byte. In the case of the present configuration, this value is equal to ~70 MB. If the second stage, i.e., the network simulation, runs on the CPU, the code allocates a total amount of memory given by Equation (16):

(16) $$\begin{array}{l} MEMcpusim=42542\;n_{goc}+12220\;n_{grc}+204{\;n}_{glo}+8004\;n_{MF} \end{array}$$

In the simulation of the present network configuration, the allocated amount of RAM is ~5 GB.

If the network simulation is performed on the GPU, the major constraint is represented by the available RAM provided on the device. In order to evaluate the maximum volume that can be reproduced with a specific board, it is important to compute the amount of global memory device to be allocated using the *cudaMalloc* function. Considering the network configuration simulated, the code allocates 580 B for parameters used both for the GOCs and GRCs activities, whose allocation is not proportional to the number of reproduced cells. On the other hand, it is necessary to allocate space for the variables used in the neuronal activity computation. This memory size is proportional to the GOCs and GRCs number and, for this reason, *2788* × *n*_*goc*_ B and *7172* × *n*_*grc*_ B are allocated, respectively. Therefore, the total amount of memory needed to reproduce a generic network configuration is given by Equation (17).

(17) $$\begin{array}{l} RAMGPU=580+2788\;n_{goc}+7172\;n_{grc} \end{array}$$

It is worth noticing that the RAM occupancy on the GPU is lower than on the CPU. The reason is that part of the connectivity is processed on the CPU; therefore, these data are not allocated on the GPU memory. Moreover, this memory amount can be generalized if two or more GPUs are used: in this case the values of *n*_*goc*_ and *n*_*grc*_ should be divided by the number of available devices.

If the number of cells is expressed as a function of cellular densities, it is possible to estimate if the volume of a certain network configuration can be stored using a specific GPU board. Equation (18) expresses the bound of the volume in function of the neurons densities and the available RAM memory.

(18) $$\begin{array}{l} V\leq \;\frac{RAMGPU-580}{2788\;n_{goc}+7172\;n_{grc}} \end{array}$$

In Equation (11), *V* is the volume expressed in mm^3^ and the memory occupancy is measured in byte. In the configuration adopted in this work, the total amount of allocated memory is ~2.88 GB, which represents the ~24% of the RAM of the NVIDIA RTX 2080 board. It is possible to conclude that the amount of memory allocated for the network design stage is negligible compared to the simulation stage performed both on the CPU and on the GPU. Finally, the memory requirements of the two stages are compatible with a standard desktop system. Therefore, it is not mandatory to use a cluster or supercomputer to run a realistic simulation with the proposed system.

### Scalability Analysis

The scalability of the proposed system has been evaluated considering two network with x and y dimensions halved (Network2) and doubled (Network3) with respect to the network described in the previous sections (Network1). The performance has been evaluated both in terms of elements placed and connected and of processing times. In terms of elements placement and connections, the considerations are the same made for the original network. Concerning the processing times, Network2 takes approximatively four times less than Network1. This is an expected value since the volume simulated in Network1 is four time the one simulated in Network2. Similarly, network3 runs four times slower than network1 as it has a quarter of the volume.

### Comparison With the State of the Art

The main differences between this work and the literature are related to the neuronal models chosen to reproduce the activity of the neurons, the simulation duration and the integration time step. Here, some of the most relevant and similar works at the state of the art are reported and compared with this work. In Naveros et al. (2015), authors developed an event- and time-driven spiking neural network simulator for a hybrid CPU-GPU platform. It consists of a very dense granular layer and a Purkinje layer with a small number of cells, where neurons are reproduced using LIF models and characterization tables (computed offline) containing the dynamic of each cell. To reproduce 10 s of neuronal activity, the simulation of 3 million neurons and 274 million synapses takes 987.44 s on an Intel i7 CPU equipped with 32 GB RAM and an NVIDIA GTX 470 GPU equipped with 1.28 GB RAM. This result cannot be directly compared to this work for two main reasons: the most important is the different model chosen and the other is related to the integration step, which varies from 0.1 to 1 ms that is higher than the one used in this work (0.025 ms).

Another interesting work that reproduces the cat cerebellum network containing more than a billion spiking neurons, is described in Yamazaki et al. (2019). Authors do not exploit the GPU technology but an HPC special purpose computer equipped with 1280 PEZY-SC processors. This system elaborates in real-time 1 s of neuronal activity, with an integration step of 1 ms. Also in this case, cells are described by LIF models and the connectivity rules are not updated. Moreover, the synapses are characterized only by the AMPA receptors. Finally, this architecture represents a completely different philosophy that from one side benefits the application specificity, from another one follows a not fairly comparable approach in terms of programmability, size/performance ratio and technological life of the employed components.

Authors of Gleeson et al. (2007) provide a tool to build, visualize and analyse network models in a 3D space. The network design reproduces very realistic and complex neuron morphologies exploiting the Hodgkin and Huxley model. Nevertheless, they run simulations of up to only 5,000 neurons on a single-processor machine that takes 1–2 h for 4 s of activity. In this case, even if the morphology is very detailed, the simulation part is not so efficient as the one proposed in this work. On the other hand, the cerebellar granular layer network developed in Solinas et al. (2010) is the one considered as reference for the present work. In fact, these networks present the same mathematical models (even if their models are written for the NEURON simulator) and connection rules. The main difference concerns the cellular morphology and the elements displacement. In this case, the cellular soma is represented by a point (not sphere) and this means that two soma can be overlapped. Moreover, during the cells displacement, the algorithm does not take into account the minimum distances between cells. They create a network inside a 3D space (i.e., a cube with 100 μm edge length) and that includes 315 MFs and 4,393 neurons (4,096 GRCs, 27 GOCs, 270 basket, and stellate cells). The reproduction of 3 s neuronal activity requires about 20 h on a Pentium-5 dual-core and 30 min using 80 CPUs on the CASPUR parallel cluster.

The work in Van Der Vlag et al. (2019) reports a multi-GPU implementation of a neuronal network based on the Hodgkin and Huxley model. The connectivity is based on the uniform or on the Gaussian distribution. Therefore, no realistic connection rules are considered. Moreover, the simulated time is only 100 ms with a time step of 0.05 ms.

Authors of Yavuz et al. (2016) proposed a systems to automatically generate CUDA kernels and runtime codes according to a user-defined network model. The work only supports single GPU systems.

A multi-GPU framework is proposed in Chou et al. (2018). However, this framework only includes the four and nine parameters Izhikevich models. Moreover, the authors evaluated the performance on a random spiking network. Thus, a direct comparison with our work would not be fair.

In Casali et al. (2019), authors present the whole cerebellar network reconstruction (i.e., granular, Purkinje, and molecular layers) based on the morphological details and connection rules used also in this work. Considering the design part of the system, the main differences with GES are the absence of the gap junctions and of the organization of the MFs in *rosettes*. Another important aspect to highlight is that in Casali et al. (2019) neurons are represented with single-point LIF models since the work is focused on a detailed network construction. Another difference between the systems is that the network in Casali et al. (2019) is simulated on pyNEST and pyNEURON while, in GES, optimized codes for the network design and simulation have been developed in C/CUDA languages. Authors in Casali et al. (2019) simulated a cerebellar cortex volume of 400 × 400 × 330 μm^3^ with a total amount of 96,734 cells even if the system is scalable. Authors do not provide information about the simulation time and the technical features of the HPC system used for the code elaboration. The integration step is set to 0.1 ms, so four times bigger than the one used in this work. Even if this network and the one described in the present work are based on the same physiological data exploited in the network reconstruction, it is not possible to make a comparison on the efficiency of the two systems since some data are missing.

### Limits and Future Works

Even if the GES system reconstructs the granular layer and reproduces its behavior, some aspects can still be improved. One of the main features of this simulator is that it is possible to change the models representing the neurons, without any modifications in the network design module. For this reason, one of the aspects that can be improved is the introduction of multi-compartment models with active compartments. This aspect will lead also to include more detailed morphologies, which will be also graphically shown through the graphical interface. Another aspect that could be improved in the design module is the introduction of a more specific constraint in the way the gap junctions are generated. Moreover, it will be interesting to reproduce a larger area of the granular layer where the MFs will stimulate more than one cluster of GLOs. Finally, since authors have already developed the Purkinje cells simulator on GPU (Torti et al., 2019), an efficient way to include these cells in the network will be studied. In this way, also the molecular and Purkinje cell layers will be reconstructed to obtain a complete cerebellar cortex network.

## Conclusions

The use of HPC technologies in computational modeling in neuroscience is becoming more and more attractive and widespread. In particular, the GPUs play a critical role in the large-scale networks elaboration where the activity of a huge number of connected neurons is reproduced.

This paper presented the GES system capable of reconstructing, simulating and visualizing the cerebellar granular layer, exploiting a desktop system with the GPU device.

The algorithm reconstructs the cerebellar granular layer following detailed rules and data aligned with the state of the art, targeting a high level of realism. The granular layer reconstruction in a 3D space is performed by an efficient serial code that takes <4 min to place and connect the neurons in a 600 × 150 × 1,200 μm^3^ volume (with 432,000 GRCs, 972 GOCs, 32,399 GLOs, and 4,051 MFs).

The simulator is also characterized by two parallel codes elaborating the network neuronal activity. The GPU device has proved to be vital to strongly reduce the computational time of the serial elaboration. Different protocols considering background, bursts and the combination of them have been tested. In particular, *Prot4* provided the most realistic scenario performing both background and bursts. In this case, the system reproduces 10 s of neuronal activity in 4.34 and 3.37 h exploiting a single and multi-GPU desktop system (equipped with one or two NVIDIA RTX 2080 GPU, respectively). Moreover, if the code runs on one node of the EOS system the processing time further decreases to 3.52 and 2.44 h exploiting one or two NVIDIA V100 GPU, respectively. The processing time of the related serial code takes ~135 h (~5.61 days) on an Intel i9 CPU and this means that the parallel versions reach a speedup up to ~38× in the single-GPU version, and up to ~55× in the multi-GPU code. This kind of technology and the development of an efficient code allowed to perform very long simulations, useful to study particular network behaviors reproducible only analyzing long time frames. In this work, authors presented a first long-lasting simulation (*Prot4*), reproducing 50 s of the network activity in ~13 h on one node of the EOS system. A crucial aspect to highlight is that the code is flexible and allows the user to reconstruct and simulate networks with different dimensions. Finally, a graphical interface has been developed to graphically analyse the results.

## Data Availability Statement

The code for this study can be found in the mclab website http://mclab.unipv.it/index.php/ges. The source code is available from the correspondent authors upon reasonable request.

## Author Contributions

GF, ET, and SM: conceputalization and methodology. GF and ET: investigation, software, and writing—original draft. SM, ED'A, and FL: writing—review and editing. FL: supervision. All authors contributed to the article and approved the submitted version.

## Conflict of Interest

The authors declare that the research was conducted in the absence of any commercial or financial relationships that could be construed as a potential conflict of interest.

## Footnotes

^1^*NVIDIA GPU Direct*. Available online at: https://developer.nvidia.com/gpudirect (accessed October 1, 2020).
